# Optimal acupuncture methods for lower limb motor dysfunction after stroke: a systematic review and network meta-analysis

**DOI:** 10.3389/fneur.2024.1415792

**Published:** 2024-07-10

**Authors:** Yaning Liu, Yuqi Tang, Linjia Wang, Pei Yu, Can Wang, Lichuan Zeng, Jing Yuan, Ling Zhao

**Affiliations:** ^1^Acupuncture and Tuina School, Chengdu University of Traditional Chinese Medicine, Chengdu, Sichuan, China; ^2^Department of Radiology, Hospital of Chengdu University of Traditional Chinese Medicine, Chengdu, Sichuan, China

**Keywords:** stroke, lower limb dysfunction, network meta-analysis, acupuncture, rehabilitation

## Abstract

**Background:**

The lower limb motor dysfunction caused by stroke is one of the main sequelae affecting patients’ ability to live normally in the later period. Acupuncture treatment of limb movement dysfunction after stroke has been recommended by authoritative guidelines for reducing limb spasticity, enhancing limb strength and so on. However, the efficacy of different acupuncture methods in treating lower limb motor dysfunction after stroke remains controversial.

**Objective:**

In this paper, network meta-analysis (NMA) was used to prioritize various acupuncture intervention combinations commonly used in clinical practice, try to screen the acupuncture intervention scheme with the highest clinical efficacy and safety, and explore its rationality in guiding clinical practice.

**Methods:**

We searched a total of 4,312 studies from 8 databases and 2 clinical trial registries, and selected 43 articles for systematic review. We used pairwise meta-analysis and network meta-analysis to evaluate the efficacy and ranking of various acupuncture interventions. At the same time, the risk of bias, publication bias, and sensitivity of included randomized controlled trials were analyzed. The main outcome indicator was Fugl-Meyer assessment of lower extremity (FMA-LE), and the secondary outcome indicators were Modified Barthel Index (MBI), Berg balance scale (BBS) and Modified Ashworth scale (MAS).

**Results:**

A total of 4,134 patients in 43 studies were included. The intervention included 9 acupuncture-related treatments, of which 20.9% were classified as high-risk. Among the four outcome indicators in pairwise meta-analysis, the effect of body acupuncture combined with conventional rehabilitation has the highest comprehensive credibility in terms of efficacy and safety comparing with conventional rehabilitation [SMD = 1.14, 95%CI (0.81, 1.46)], [SMD = 1.35, 95%CI (0.97, 1.72)], [SMD = 1.22, 95%CI (0.39, 2.05)], [SMD = 1.21, 95%CI (0.74, 1.44)]. In addition, multiple intervention methods, for example, warm acupuncture plus rehabilitation treatment for MBI and electroacupuncture plus body acupuncture plus rehabilitation treatment for BBS, may increase certain additional effects on different outcome indicators.

**Conclusion:**

This study proves that body acupuncture combined with rehabilitation treatment is the most widely used intervention method with the highest evidence quality in the treatment of lower limb motor dysfunction after stroke. However, for some other acupuncture methods, large samples and high-quality clinical randomized controlled trials are still needed to be fully verified.

## Introduction

Stroke remains one of the most serious global public health events at present (1). According to the latest neurological epidemiological survey, stroke is the leading cause of death and disability among non-communicable neurological diseases (2, 3). Lower limb motor dysfunction is one of the most common complications after stroke. Correlational studies showed that about 40–60% of patients have different degrees of lower limb motor dysfunction after stroke (4, 5). Walking and mobility problems are a major cause of long-term disability after stroke, which poses a great challenge for patients to recover walking ability and live independently after the onset of stroke. Therefore, improving motor function and reducing long-term disability is the main therapeutic goal of post-stroke rehabilitation.

At present, many rehabilitation methods that promotes neural recovery through multiple stimulation have been widely used in clinical practice, such as Rood, Bobath, Brunnstrom, Proprioceptive Neuromuscular Facilitation (PNF) (6), Electrical stimulation (7) and Extracorporeal shock wave therapy (8) etc. However, substantial progress remains to be made in stroke rehabilitation practice to meet the growing demand for its services. The efficacy of some rehabilitation methods has not reached the ideal clinical efficacy, and different patients have different degrees of response to various rehabilitation methods (9). Therefore, how to choose an appropriate and effective way of rehabilitation based on traditional treatment methods has become an urgent problem to be solved.

Acupuncture, as a non-drug therapy with a long history, has been widely used in China and many other countries for the recovery of various sequelae after stroke, including limb dysfunction, swallowing dysfunction, depression, urinary retention and so on (10–12). A review study from Cochrane Library suggests that acupuncture may have potential benefits in improving neurological deficits after stroke (13). Meanwhile, a meta-analysis found that acupuncture combined with conventional treatment has more advantages in promoting the recovery of upper limb motor function than rehabilitation alone (14). However, there is no high quality literature at present to comprehensively evaluate the efficacy and safety of various acupuncture methods in the intervention of lower limb dysfunction after stroke. Therefore, this study attempts to comprehensively collect relevant clinical evidence and conduct systematic analysis to screen out the relatively optimal clinical acupuncture-related interventions.

In this paper, the NMA method was used to combine various types of direct and indirect evidence from RCTs to form a randomized controlled trial network comparing different combination of acupuncture treatments (15). Using this method to prioritize different acupuncture schemes, screen the best acupuncture treatment for lower limb motor dysfunction after stroke, and explore the rationality of its clinical guidance.

## Materials and methods

The study design followed the PRISMA-NMA guideline (16) (Supplementary File S1) and a version of this study was registered on Open Science Framework (Registration DOI:10.17605/OSF.IO/3ZVNU).

### Search strategy

To obtain a sufficient number of references, we searched major English and Chinese databases and clinical trial registries from the establishment of the database to November 19, 2023. The search database includes: China National Knowledge Infrastructure (CNKI), WANFANG Database (WF), VIP Database for Chinese Technical Periodicals (VIP), Chinese biomedical literature service system (SinoMed), PubMed, Web of Science (WOS), Cochrane Library and Embase. The clinical trial registries included the Chinese Clinical Trial Registry (ChiCTR) and the International Standard Randomized Controlled Trial Number (ISRCTN) Register. In addition, MeSH terms and free words used in this study include: (1) Acupuncture, electroacupuncture, fire acupuncture, ear acupuncture, warm acupuncture, acupoint, etc., (2) stroke, cerebral infarction, cerebral hemorrhage, etc., (3) paralysis, spasm, motor dysfunction, etc. (4) lower limbs, legs, knees, feet, etc. and (5) clinical trials, randomized controlled trials, etc. Taking Pubmed as an example, the specific search strategy is shown in Table 1.

**Table 1:** Search strategy for PubMed database.

Steps	Search
#1	“Acupuncture therapy”[MeSH Terms] OR “Acupuncture”[MeSH Terms] OR Acupuncture, Ear[MeSH Terms] OR “meridian*”[Title/Abstract] OR “acupoint*”[Title/Abstract] OR “warm needling”[Title/Abstract] OR “warm acupuncture”[Title/Abstract] OR “electronic acupuncture”[Title/Abstract] OR “electro-acupuncture”[Title/Abstract] OR “electroacupuncture”[Title/Abstract] OR “fire acupuncture”[Title/Abstract] OR “scalp needle”[Title/Abstract] OR “abdominal needle”[Title/Abstract] OR “wrist ankle needle”[Title/Abstract] OR “triple puncture”[Title/Abstract] OR “dry needle”[Title/Abstract] OR “needle”[Title/Abstract] OR “body acupuncture”[Title/Abstract] OR “manual-acupuncture”[Title/Abstract]
#2	“clinical trials as topic”[MeSH Terms] OR “random allocation”[MeSH Terms] OR “therapeutic use”[MeSH Subheading] OR “clinical”[Title/Abstract] OR “trial”[Title/Abstract] OR “clinical trial”[Publication Type] OR “random*”[Title/Abstract]
#3	“Stroke”[MeSH Terms] OR “Cerebrovascular Accident”[Title/Abstract] OR “CVA”[Title/Abstract] OR “Brain Vascular Accident”[Title/Abstract] OR “Apoplexy”[Title/Abstract]
#4	“Hemiplegia”[MeSH Terms] OR “Paralysis”[MeSH Terms] OR “motor function”[Title/Abstract] OR “Dysfunction”[Title/Abstract]
#5	“Lower limbs”[Title/Abstract] OR “lower extremities”[Title/Abstract] OR “leg”[Title/Abstract] OR “digit”[Title/Abstract] OR “toe”[Title/Abstract] OR “knee”[Title/Abstract] OR “ankle”[Title/Abstract] OR “foot”[Title/Abstract] OR “thigh”[Title/Abstract] OR “Lower limb”[Title/Abstract] OR “Lower extremity”[Title/Abstract]
#6	#4 OR #5
#7	#1 AND #2 AND #3 AND #6

### Inclusion and exclusion criteria

#### Inclusion criteria

(P) Patients with a first ischemic or hemorrhagic stroke diagnosed according to international criteria with lower extremity motor dysfunction; over the age of 18, regardless of gender or nationality;

(I) Received acupuncture treatment (body acupuncture, electroacupuncture, scalp needle, warm acupuncture, fire acupuncture, abdominal needle, eyes acupuncture, acupotome, wrist ankle needle, etc.) and the non-acupuncture recommended primary stroke rehabilitation treatments received needed to be consistent with the control group;

(C) Acupuncture treatment or non-acupuncture interventions (fake acupuncture or recommended primary stroke treatment such as surgery, medication, rehabilitation or symptomatic supportive care);

(O) Outcome measures included at least Fugl-Meyer assessment of lower extremity (FMA-LE)/ Modified Barthel Index (MBI)/Berg balance scale (BBS)/Modified Ashworth scale (MAS);

(S) Randomized controlled trials (excluding non-randomized controlled trials such as systematic reviews, case reports, conference abstracts, clinical experience and animal trials); However, this study included a clinical research type of dissertation.

#### Exclusion criteria

(1) Studies with repeated literature or secondary analysis, for duplicates, choose to keep the most up-to-date and comprehensive literature;
(2) Non-randomized controlled trials (including animal studies, books, conference abstracts, protocols, communications, case reports, reviews, and systematic reviews);
(3) Non-post-stroke lower extremity motor dysfunction;
(4) Acupuncture therapy was not used in the intervention group;
(5) The intervention group or control group used Traditional Chinese medicine or Chinese medicine decoction pieces;
(6) The result index does not match.

### Study selection and data extraction

Firstly, we used the above search strategy to retrieve relevant studies from the database (CNKI, WF, VIP, SinoMed, WOS, PubMed, Embase and Cochrane Library) and clinical trial registry (ISRCTN and ChiCTR) and import them into the NoteExpress (V3.7.0.9258) database. In this process, two authors (CW and PY) independently filter articles from several databases to increase the confidence of the results. In case of disagreement between authors, judgment was left to the third author (YT).

Two researchers (YL and LW) independently extracted relevant data from the literature into Microsoft Excel 2016. It included literature name, author, country, stroke type, stroke stage, Brunnstrom stage, disease course, sample size, sample ratio, age, sex ratio, randomization method, blind method, distribution hiding, intervention, outcome index, outcome, adverse events, acupuncture point and duration, etc. After completion of the data extraction, cross check by two researchers, and the third researchers are discussed, in order to reduce the differences in the process of data extraction. In cases where the data is unclear, we will contact the first or corresponding author of the paper via email to ask for further information about the data.

### Risk of bias assessment

Two investigators used the Cochrane Collaboration risk of bias tool (17) to independently assess the risk of bias in included RCTs. Assessment items included: (1) random sequence generation; (2) allocation concealment; (3) blinding of participants and personnel; (4) blinding of outcome assessment; (5) incomplete outcome data; (6) selective reporting; (7) other bias. All of the above biases were assessed and classified into low risk, high risk, and undefined risk. If disagreements arise during the evaluation, a third investigator is consulted. A trial is considered low risk if all of its assessed items are low risk, or if there are fewer than three items of undefined risk. A trial is rated as high risk if two or more items in the trial are assessed as high risk. Other trials were classified as having uncertain risks.

### Data analysis

Data analysis mainly used Review Manager software (Revman 5.3), STATA software (V16.0) and Aggregate Data Drug Information System (ADDIS) software (V1.16.8). Direct comparisons were made using a pairwise meta-analysis. Standardized mean differences (SMD) and 95% confidence intervals (CIs) represent continuous outcomes. A fixed effects model was used for overall analysis in the absence of obvious heterogeneity (I2 < 50%); otherwise, a random effects model was used. We used a Markov chain Monte Carlo method to conduct NMA using the ADDIS. Subsequently, we generated various NMA plots in Stata software (Version 16.0 MP). Moreover, the node-splitting method was used to divide the evidence of each comparison into direct evidence and indirect evidence. The surface under the cumulative ranking curve (SUCRA) was used to rank the advantages and disadvantages of different Acupuncture related treatments.

### Publication bias assessment

Publication bias was assessed using funnel plots generated by Stata software.

### Sensitivity analysis

We evaluated the robustness of each result by sensitivity analysis and excluded high-risk bias studies.

## Results

### Study identification and selection

**Figure 1:**
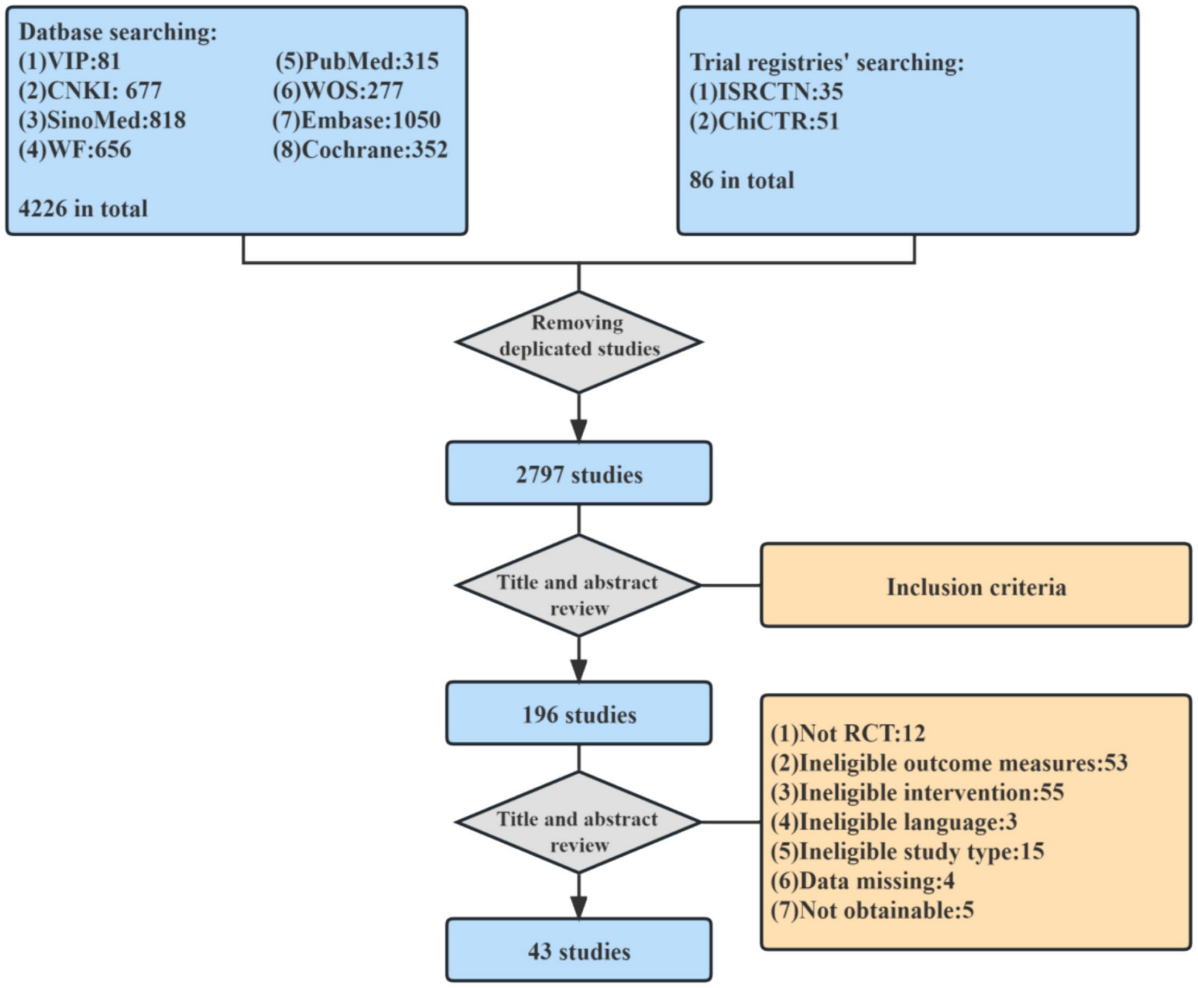
Flow chart of study selection.

A total of 4,226 literatures were retrieved from 8 databases and 2 clinical trial registries. A total of 236 studies that basically met the inclusion criteria were initially screened out by two reviewers based on their titles and abstracts. The other two reviewers reviewed the full text of the preliminary screening literature, and finally selected 43 literatures (18–60) that fully met the criteria to be included in this meta-analysis (Figure 1).

### Description of study inclusion

We finally selected 43 RCTS (18–60), including a total of 4,134 stroke patients, of which 30 patients dropped out during the trial through literature search and data screening. All included studies detailed in Supplementary File S2. Most of the included studies were from China (42/43) (18–56, 58–60), and only one was from the United States (57). Stroke types were clearly described in 36 studies (19, 21–31, 33–37, 39–42, 44–46, 48–50, 52–60), including 30 studies (19, 21, 22, 24–29, 31, 35–37, 39, 40, 42, 44–46, 48–50, 52–54, 56–60) involving both ischemic and hemorrhagic, five studies (23, 30, 33, 41, 55) involving pure ischemic stroke, and one study (34) involving pure hemorrhagic stroke. There are 13 papers (19, 20, 22, 26, 28, 29, 31, 34, 39, 40, 43, 47, 59) describe Brunnstrom stages and 33 papers (18–20, 24–26, 28, 29, 31–50, 52–54, 59, 60) document the course of the disease after stroke. The allocation ratio was 1:1 for all but two studies (35, 43), which had a 1:1:1 allocation ratio. In addition, interventions, outcome measures, treatment cycles, and outcomes were fully documented for all studies.

A total of 16 different acupuncture combinations were included in the study, as well as conventional drugs and rehabilitation treatments. Conventional drugs and rehabilitation treatment (RT) were used in all clinical trials. The most frequently used acupuncture scheme was body acupuncture and rehabilitation treatment (BA+RT, 13), followed by electroacupuncture and rehabilitation treatment (EA + RT, 6), electroacupuncture and body acupuncture and rehabilitation treatment (EA + BA+RT, 6) and so on. The study subjects were 40.98% female. The most commonly used acupoints were Yanglingquan (19), Zusanli (19), Sanyinjiao (17) and Taichong (12). Among them, 7 studies were based on Xingnao Kaiqiao acupuncture method for acupoint matching.

### Assessment of risk of bias

**Figure 2:**
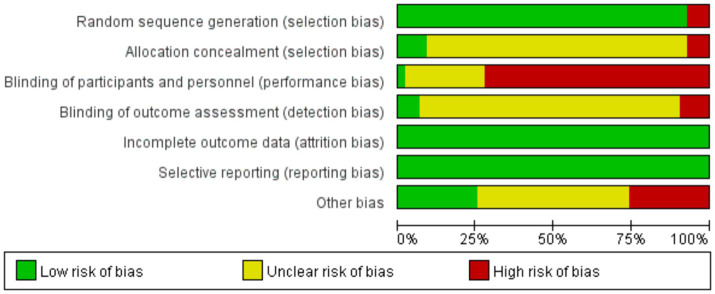
Risk of bias graph.

**Figure 3:**
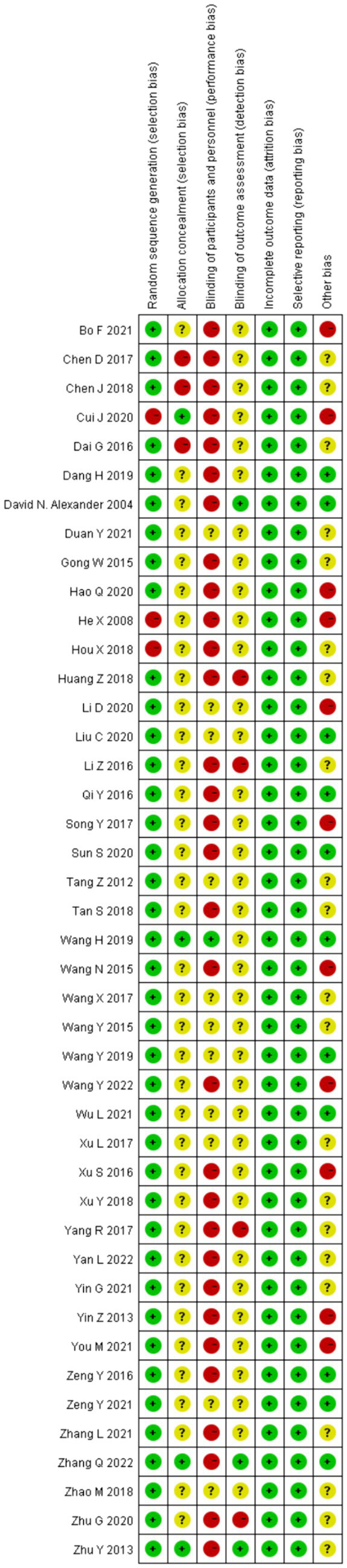
Risk of bias summary.

The risk of bias assessment results are shown in Figures 2, 3, and the detailed assessment results are shown in Supplementary File S3. Results showed that three of the 43 trials (32, 38, 56) was classified as high risk without randomization, because of the RCTs did not describe the allocation. There is ambiguity in the description of three trials (36, 45, 60) in relation to allocation concealment, which may have been knowingly predicted or adjusted. Four trials (24, 27, 44, 49) were rated as high risk in terms of blinding of outcome assessment because their assessors may have been aware of the intervention subgroups. Due to the acupuncture and conventional rehabilitation contrast is difficult to set a blind, 31 trials (18, 21–25, 27, 28, 30, 32, 33, 36, 38–40, 42, 44–50, 52–54, 56–60) was classified as high risk because of improper blinding. Eleven trials (18, 21, 22, 28, 29, 32, 42, 47, 50, 54, 56) had other high risks of bias, five trials (21, 22, 29, 50, 54) had an unbalanced ratio of stroke types between the two groups, and three trials (28, 42, 47) had large differences in the gender ratio of patients. In total, 18 trials (18, 21, 22, 24, 27, 28, 32, 36, 38, 42, 44, 45, 47, 49, 50, 54, 56, 60) were classified as having a high risk of overall bias (41.9%).

### Pairwise meta-analysis

This study conducted a Pairwise Meta-Analysis of the included randomized controlled trials. The results of the study are in Supplementary File S4. We summarize the four outcome indicators as shown in Table 2, and the combination results meaningful intervention was marked. As can be seen from the table, RT was used as the comparison object in more than half of RCTs, and most acupuncture methods were superior to RT. Meanwhile, some comparisons showed different results under different outcome indicators. For example, SN + EA + RT vs. RT showed no significant difference in FMA-LE, but showed opposite results in MBI and MAS. In addition, intervention BA+RT had the largest number of RCTs compared with other acupuncture methods, but most acupuncture intervention methods except for BA and RT were seems better than BA+RT in terms of the four outcome indicators.

**Table 2:** The results of pairwise meta-analysis.

Outcome measure	Comparison	Number	SMD (95% CI)	I^2^(%)	*P*
FMA-LE	WA+AN+RT vs RT EA+BA+RT vs EyA+EA+BA+RT **EA+BA+RT vs BA+RT** **WA+RT vs RT** **WA+SN+RT vs RT** SN+EA+RT vs RT **BA+RT vs FA+RT** **BA+RT vs AN+EA+BA+RT** **BA+RT vs BA** **BA+RT vs RT** **EA+RT vs FA+RT** **EA+RT vs RT** **FA+RT vs RT** **SN+BA+RT vs RT** **NN+RT vs RT** **EA+BA+SN+RT vs RT** **BA vs RT**	1 1 **1** **2** **1** 1 **1** **1** **1** **10** **2** **5** **2** **5** **1** **1** **1**	0.82(0.45, 1.20) −0.26(−0.63, 0.12) **0.85(0.50, 1.21)** **1.08(0.53, 1.63)** **0.79(0.33, 1.25)** 0.50(−0.02, 1.01) **−1.55(−2.13, −0.97)** **−0.47(−0.84, −0.10)** **1.20(0.80, 1.60)** **1.14(0.81, 1.46)** **−1.12(−1.53, −0.71)** **1.06(0.42, 1.70)** **1.05(0.72, 1.39)** **0.72(0.18, 1.27)** **0.87(0.34, 1.40)** **0.45(0.04, 0.85)** **−0.14(−0.51, 0.23)**	– – – 58% – – – – – 79% 33% 93% 66% 87% – – –	– – – 0.12 – – – – – *P* < 0.00001 *P* = 0.22 *P* < 0.00001 *P* = 0.09 *P* < 0.00001 – – –
MBI	**WA+AN+RT vs RT** **WA+RT vs EA+RT** **WA+RT vs RT** **WA+SN+RT vs RT** **SN+EA+RT vs RT** **BA+RT vs AN+EA+BA+RT** **BA+RT vs BA** **BA+RT vs RT** EA+RT vs NN+RT **EA+RT vs RT** SN+BA+RT vs RT BA vs RT RT vs SN+WAN+RT	**1** **1** **1** **1** **1** **1** **1** **6** 1 **1** 3 1 1	**1.23(0.84, 1.62)** **1.72(1.30, 2.14)** **0.85(0.36, 1.33)** **0.71(0.26, 1.16)** **0.58(0.06, 1.10)** **−1.19(−1.58, −0.79)** **1.46(0.89, 2.03)** **1.35(0.97, 1.72)** −0.03(−0.42, 0.36) **1.03(0.54, 1.52)** 0.20(−0.01, 0.41) −0.35(−0.86, 0.16) −0.53(−1.07, 0.02)	– – – – – – – 74% – – 0% – –	– – – – – – – *P* = 0.002 – – *P* = 0.43 – –
MAS	**WA+RT vs EA+RT** **SN+EA+RT vs RT** BA+RT vs EA+RT **BA+RT vs FA+BA+RT** **BA+RT vs BA** **BA+RT vs RT** EA+RT vs RT **SN+BA+RT vs RT** BA vs RT	**1** **1** 1 **1** **1** **3** 1 **1** 1	**0.82(0.45, 1, 19)** **0.55(0.04, 1.07)** −0.08(−0.52, 0.37) **−0.86(−1.39, −0.33)** **1.26(0.70, 1.82)** **1.22(0.39, 2.05)** 0.45(−0.02, 0.92) **0.68(0.16, 1.21)** 0.11(−0.40, 0.61)	– – – – – 89% – – –	– – – – – *P* = 0.0002 – – –
BBS	**EA+BA+RT vs BA+RT** **BA+RT vs RT** **EA+RT vs RT**	**1** **6** **2**	**0.93(0.58, 1.29)** **1.21(0.63, 1.80)** **1.09(0.74, 1.44)**	– 89% 93%	– *P* < 0.00001 *P* = 0.0001

The bold font indicates a statistical difference. WA, Warm Acupuncture; AN, Abdominal Needle; RT, Rehabilitation treatment; BA, Body Acupuncture; EA, Electroacupuncture; FA, Fire Acupuncture; SN, Scalp Needle; EyA, Eyes Acupuncture; NN, acupotome; WAN, wrist ankle needle; FMA-LE, Fugl-Meyer Assessment for Lower Extremity; MBI, Modified Barthel Index; MAS, Modified Ashworth Scale; BBS, Berg Balance Scale.

### Network meta-analysis

**Figure 4:**
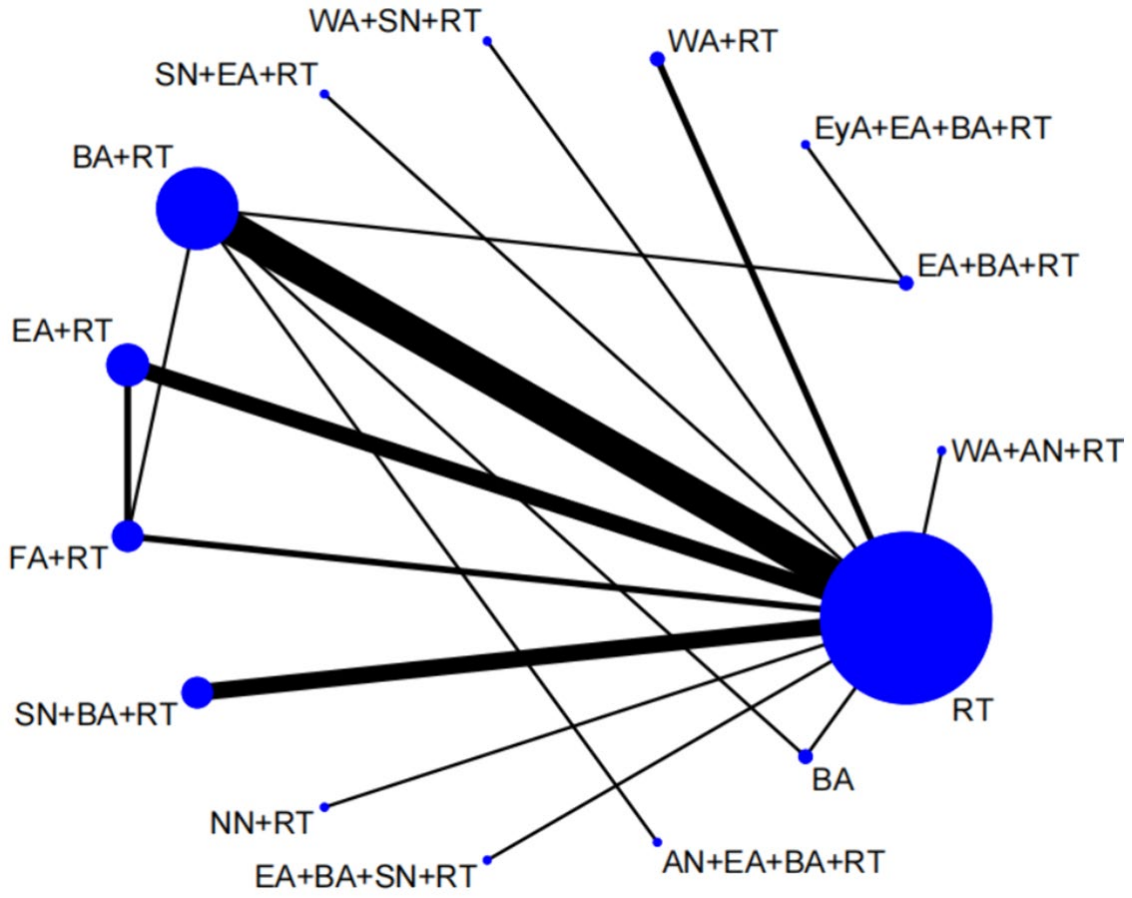
Network plot of FMA-LE. WA, warm acupuncture; AN, abdominal needle; RT, rehabilitation treatment; BA, body acupuncture; EA, electroacupuncture; FA, fire acupuncture; SN, scalp needle; EyA, eyes acupuncture; NN, acupotome.

**Figure 5:**
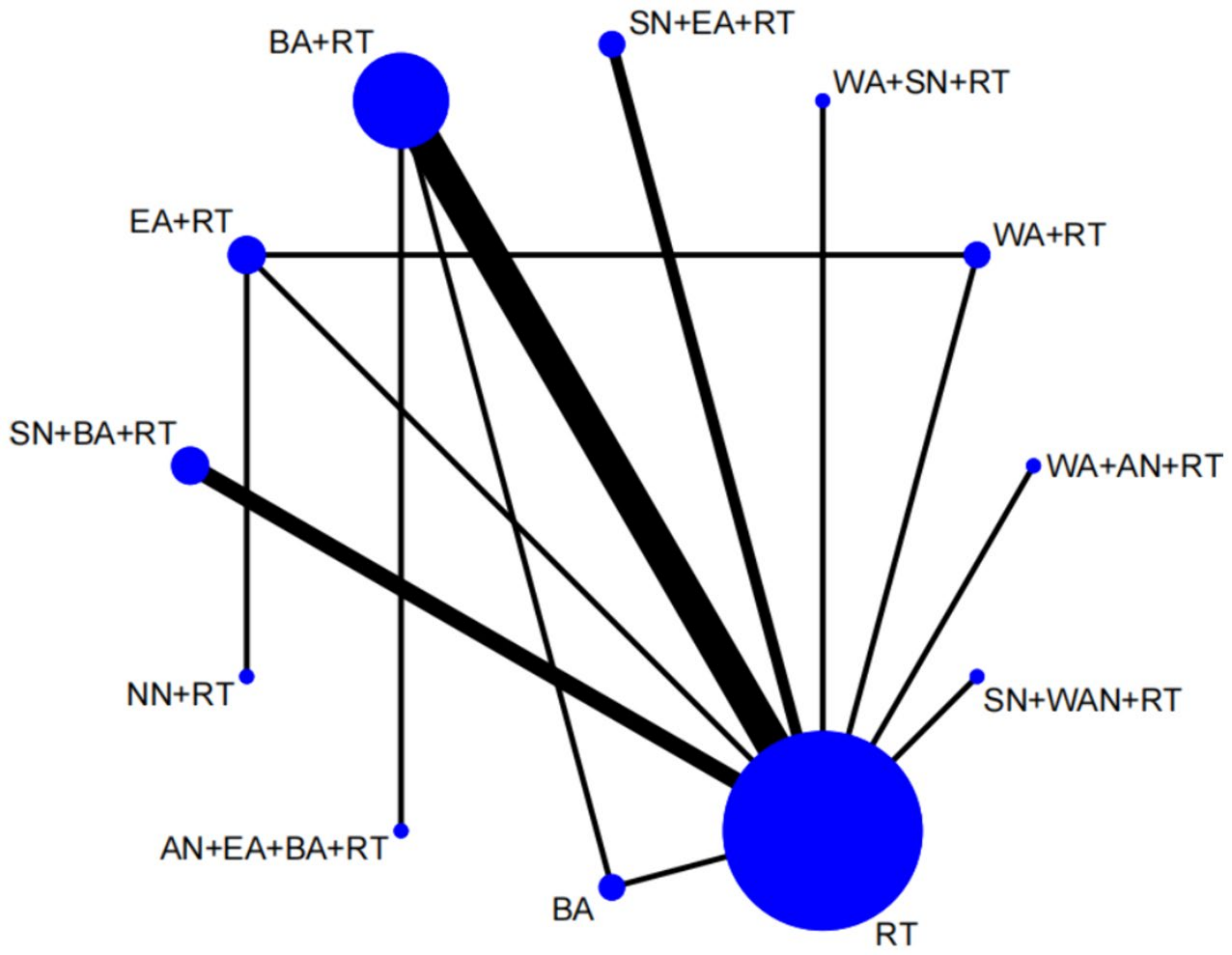
Network plot of MBI. WA, warm acupuncture; AN, abdominal needle; RT, rehabilitation treatment; BA, body acupuncture; EA, electroacupuncture; SN, scalp needle; NN, acupotome; WAN, wrist ankle needle.

**Figure 6:**
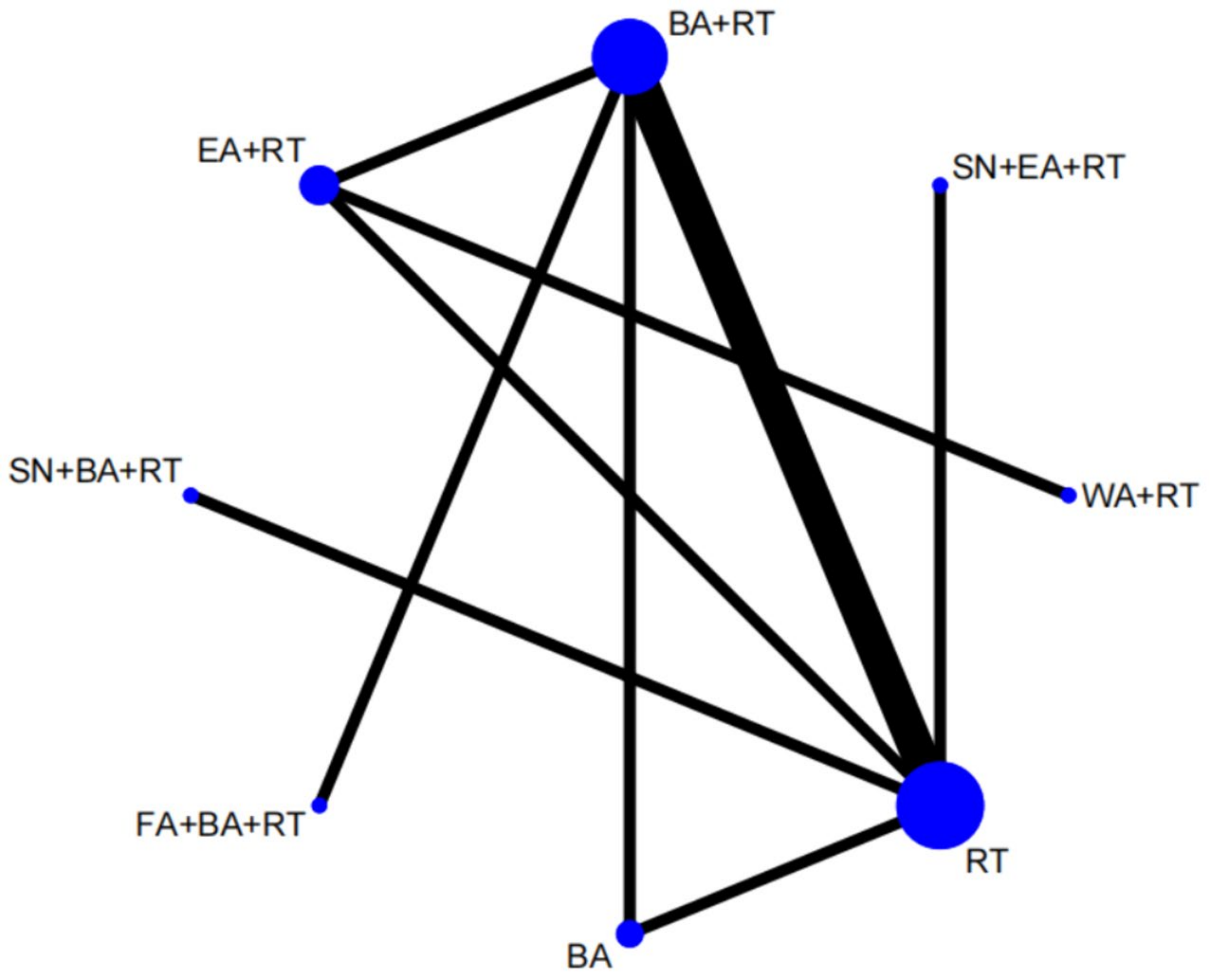
Network plot of MAS. WA, warm acupuncture; RT, rehabilitation treatment; BA, body acupuncture; EA, electroacupuncture; FA, fire acupuncture; SN, scalp needle.

**Figure 7:**
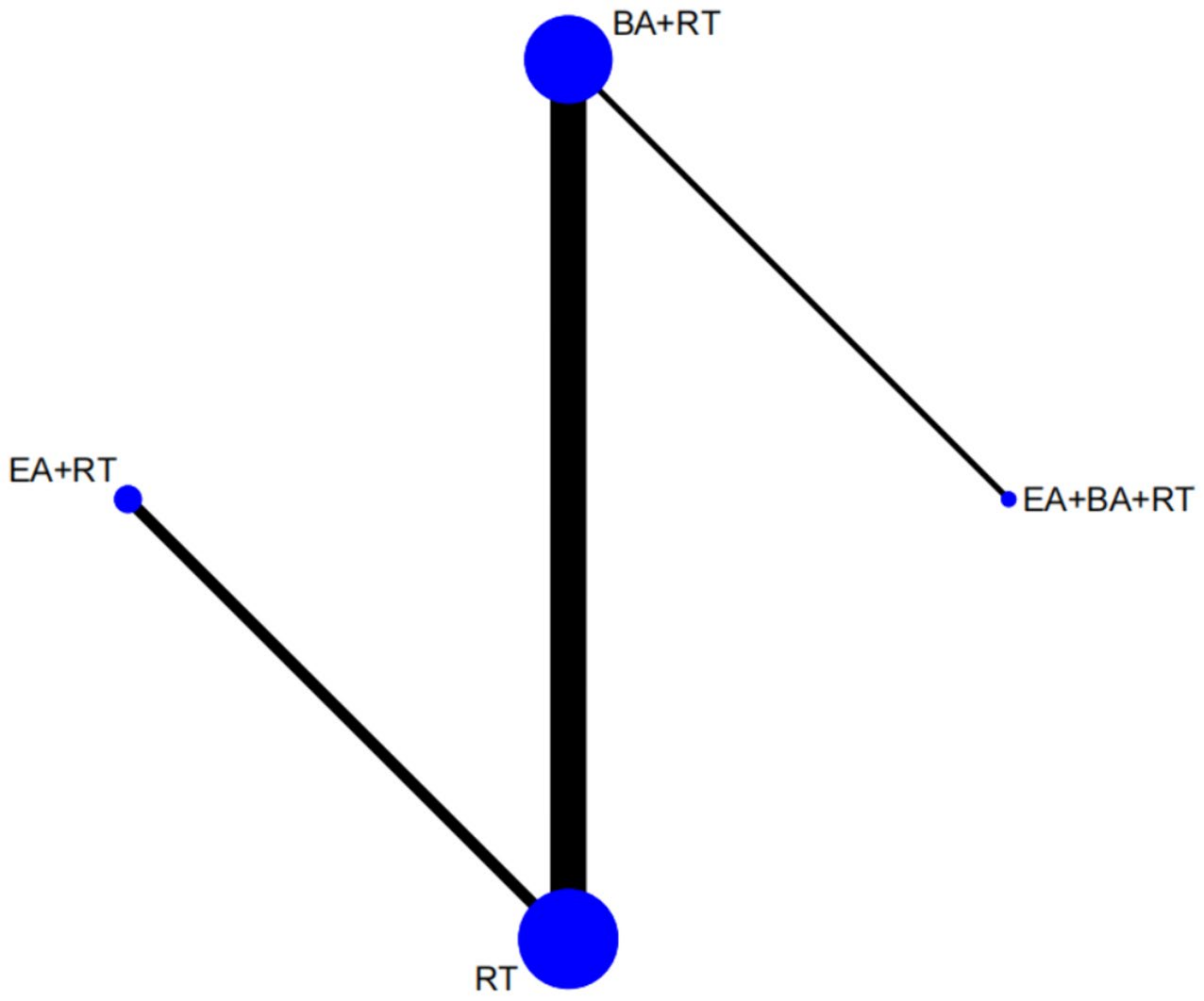
Network plot of BBS. RT, rehabilitation treatment; BA, body acupuncture; EA, electroacupuncture.

We plotted a network of different interventions for four outcome measures (Figures 4–7). The node size represents the total number of patients with RCTs included in the intervention, and the line segment thickness between the two nodes represents the total number of RCTS included in both interventions. Specifically, FMA-LE was an outcome measure for 35 trials involving a total of 3,412 patients; MBI was the outcome measure of 19 trials involving 1,770 patients; MAS was the outcome measure of 737 patients in 9 trials. BBS was the outcome measure of 9 trials involving a total of 843 patients.

As shown in Figures 4, 5, the network structure formed between the two outcome measures of FMA-LE and MBI was roughly the same, indicating that FMA-LE and MBI were commonly used outcome measures to evaluate the treatment effect of lower limb motor dysfunction after stroke. As can be seen from Figures 6, 7, the number of patients and trials using MAS and BBS as outcome indicators were relatively small. The network structure of the four outcome indicators showed that BA+RT was the most commonly used combination method in acupuncture intervention, followed by EA + RT. The comparison between BA+RT and RT are most studied. In addition, in a study34 with MAS as the outcome indicator (SN + BA+RT vs. RT), the researchers differentiated the score of the lower extremity according to the knee and the ankle, the difference between the results of the two parts was averaged during data processing.

The validity of the NMA results depends on the internal consistency of the evidence network: that is, the sources of direct and various indirect evidence should be consistent (61). In this study, the segmented node method was used to test the inconsistency in the NMA (Supplementary File S5). The results show that the direct or indirect comparisons of each segment node are not statistically significant (*p* > 0.05), indicating that there is no evidence of design inconsistency. We also tested the convergence of the model with a potential size reduction factor of 1 (Supplementary File S6).

**Figure 8:**
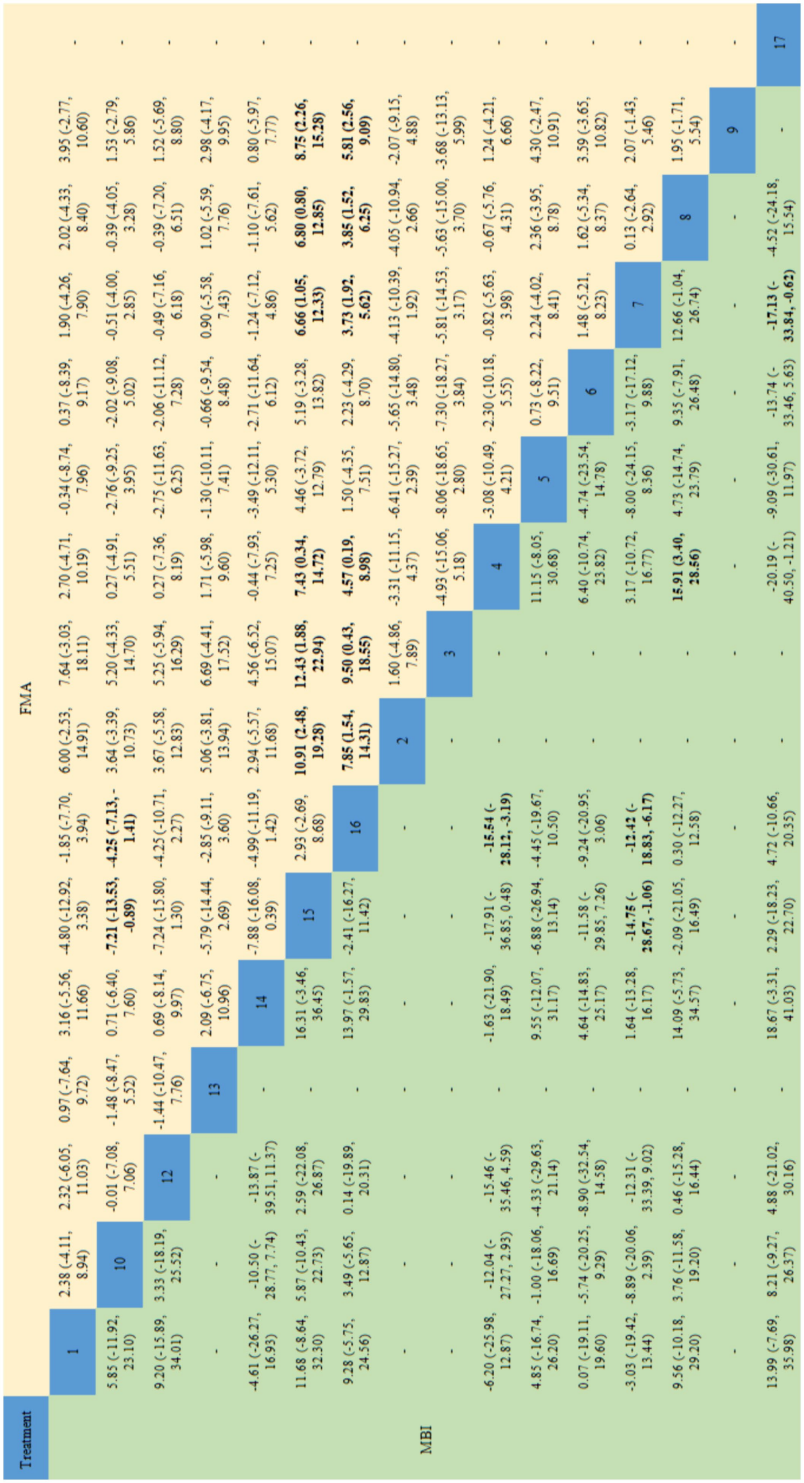
Network meta-analysis results for FMA-LE and MBI. The bold font indicates a statistical difference. FMA-LE, Fugl-Meyer Assessment for Lower Extremity; MBI, Modified Barthel Index; 1 = Warm Acupuncture+Abdominal Needle+Rehabilitation Treatment; 2 = Electroacupuncture+Body Acupuncture+Rehabilitation Treatment; 3 = Eyes Acupuncture+Electroacupuncture+Body Acupuncture+Rehabilitation Treatment; 4 = Warm Acupuncture+Rehabilitation Treatment; 5 = Warm Acupuncture+Scalp Needle+Rehabilitation Treatment; 6 = Scalp Needle+Electroacupuncture+Rehabilitation Treatment; 7 = Body Acupuncture+Rehabilitation Treatment; 8 = Electroacupuncture+Rehabilitation Treatment; 9 = Fire Acupuncture+Rehabilitation Treatment; 10 = Scalp Needle+Body Acupuncture+Rehabilitation Treatment; 12 = acupotome+Rehabilitation Treatment; 13 = Electroacupuncture+Body Acupuncture+Scalp Needle+Rehabilitation Treatment; 14 = Abdominal Needle+Electroacupuncture+Body Acupuncture+Rehabilitation Treatment; 15 = Body Acupuncture; 16 = Rehabilitation Treatment; 17 = Scalp Needle+Wrist Ankle Needle+Rehabilitation Treatment.

**Figure 9:**
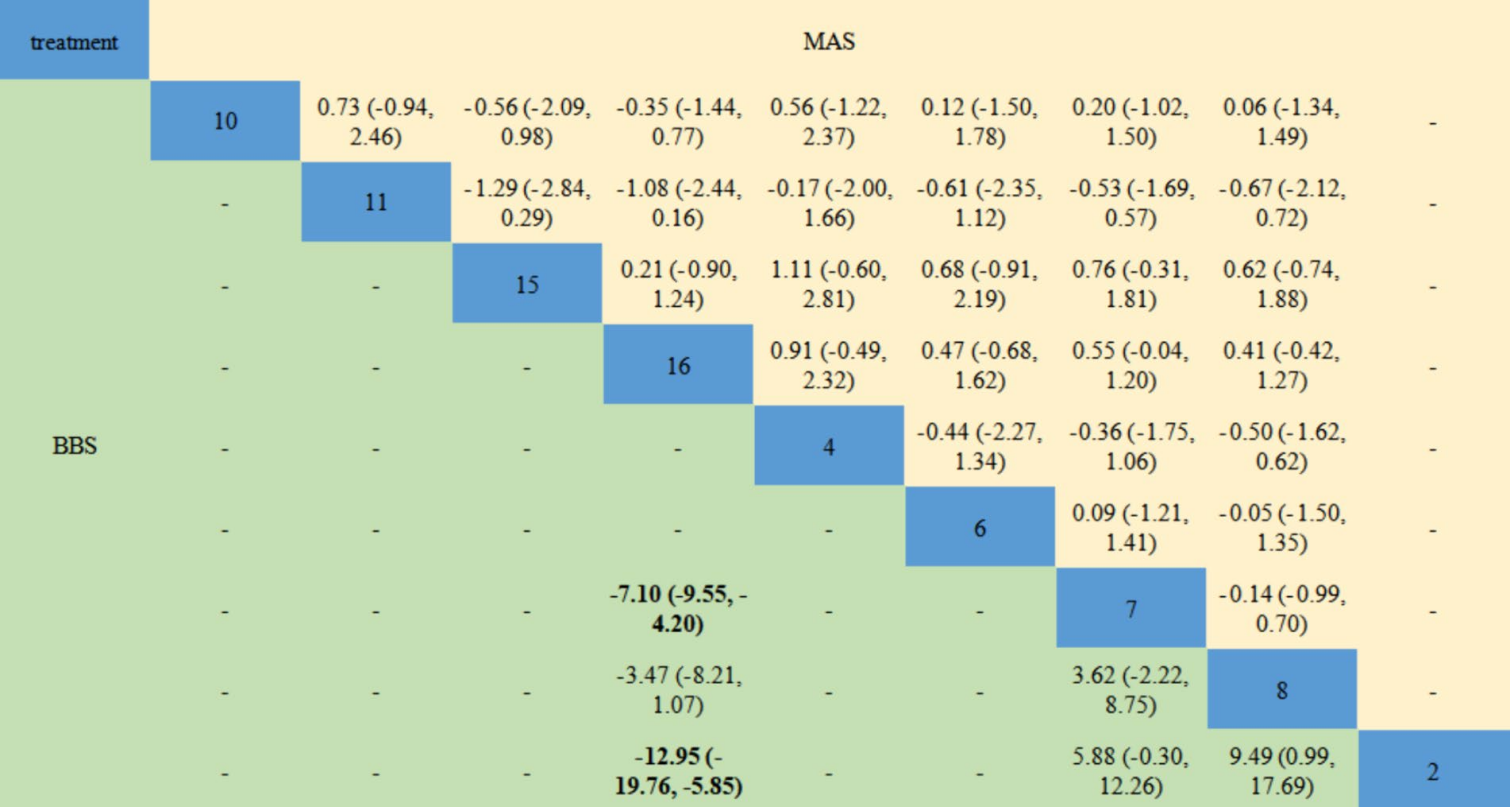
Network meta-analysis results for MAS and BBS. The bold font indicates a statistical difference. MAS, Modified Ashworth Scale; BBS, Berg Balance Scale. 2 = Electroacupuncture+Body Acupuncture+Rehabilitation Treatment; 4 = Warm Acupuncture+Rehabilitation Treatment; 6 = Scalp Needle+Electroacupuncture+Rehabilitation Treatment; 7 = Body Acupuncture+Rehabilitation Treatment; 8 = Electroacupuncture+Rehabilitation Treatment; 10 = Scalp Needle+Body Acupuncture+Rehabilitation Treatment; 11 = Fire Acupuncture+Body Acupuncture+Rehabilitation Treatment; 15 = Body Acupuncture; 16 = Rehabilitation Treatment.

Figures 8, 9 shows the NMA results. As shown in the figure, the main difference in the improvement of FMA-LE score is the comparison of acupuncture related interventions with BA(15) and RT(16), and half of the interventions are better than these two schemes. Similarly, in terms of improving MBI, the intervention combinations of WA + RT(4), BA+RT(7) and EA + RT(8) were better than BA(15) and RT(16) to a certain degree, and SN + WAN + RT(17) was better than BA+RT(7). In terms of improving BBS, EA + BA+RT(2) and BA+RT(7) are better than BA(15) and RT(16).

**Figure 10:**
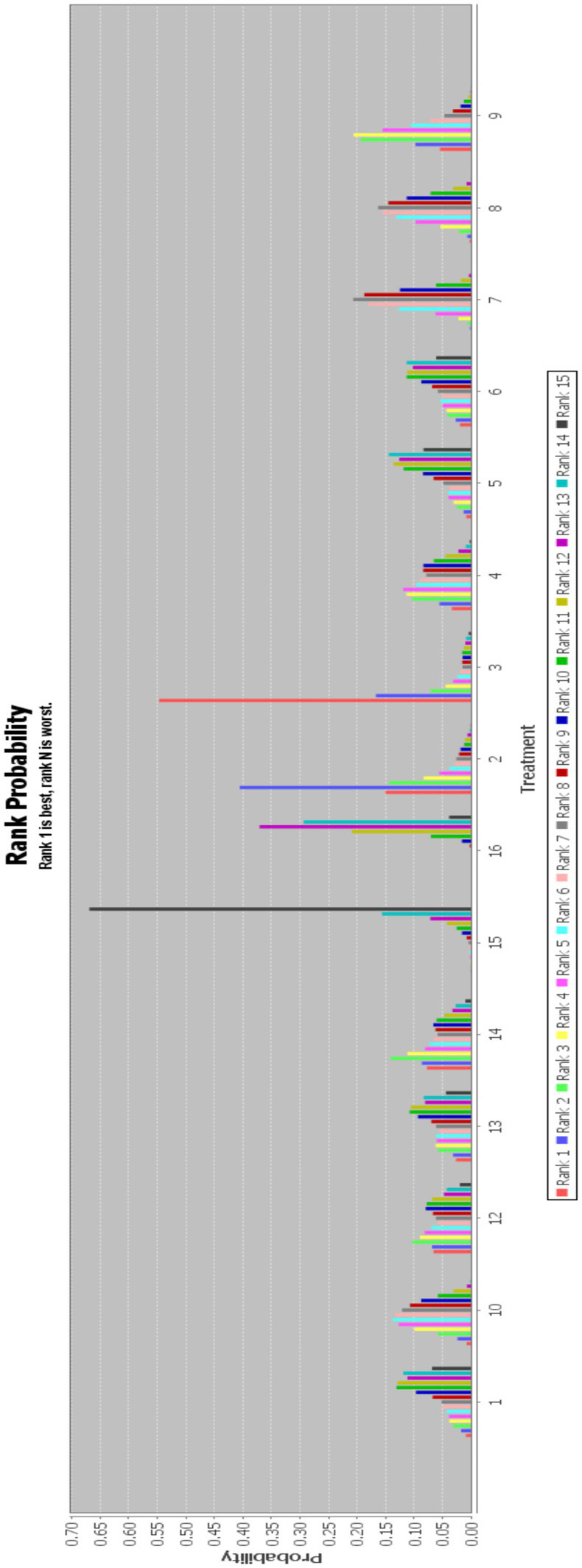
Ranking probability figure for FMA-LE. 1 = Warm Acupuncture+Abdominal Needle+Rehabilitation Treatment; 2 = Electroacupuncture+Body Acupuncture+Rehabilitation Treatment; 3 = Eyes Acupuncture+Electroacupuncture+Body Acupuncture+Rehabilitation Treatment; 4 = Warm Acupuncture+Rehabilitation Treatment; 5 = Warm Acupuncture+Scalp Needle+Rehabilitation Treatment; 6 = Scalp Needle+Electroacupuncture+Rehabilitation Treatment; 7 = Body Acupuncture+Rehabilitation Treatment; 8 = Electroacupuncture+Rehabilitation Treatment; 9 = Fire Acupuncture+Rehabilitation Treatment; 10 = Scalp Needle+Body Acupuncture+Rehabilitation Treatment; 12 = acupotome+Rehabilitation Treatment; 13 = Electroacupuncture+Body Acupuncture+Scalp Needle+Rehabilitation Treatment; 14 = Abdominal Needle+Electroacupuncture+Body Acupuncture+Rehabilitation Treatment; 15 = Body Acupuncture; 16 = Rehabilitation Treatment; 17 = Scalp Needle+Wrist Ankle Needle+Rehabilitation Treatment.

**Figure 11:**
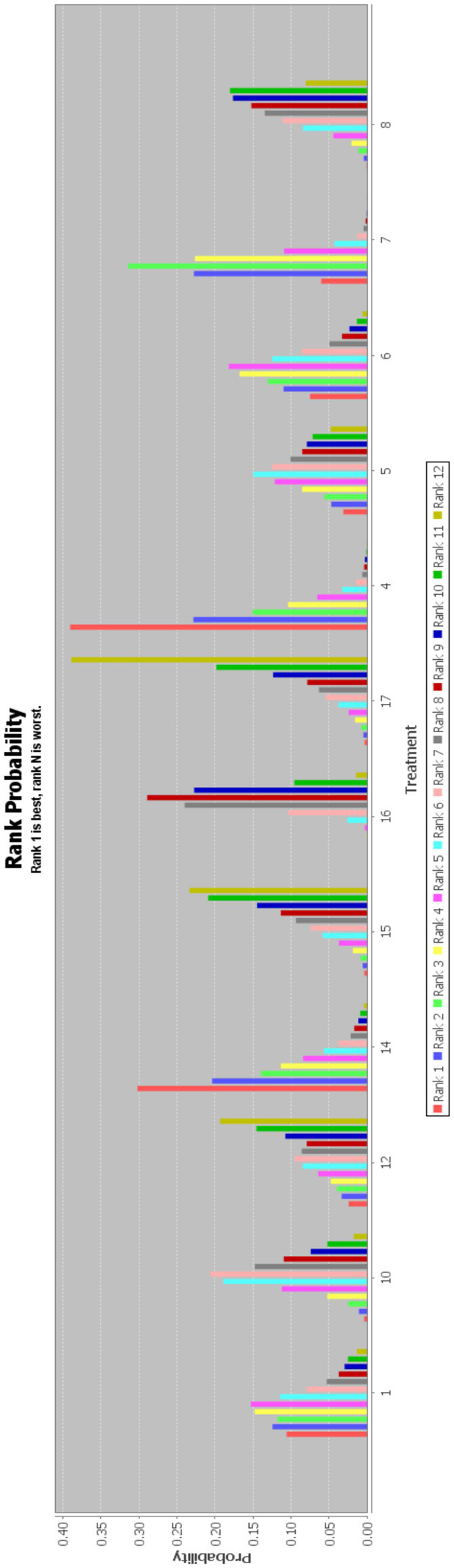
Ranking probability figure for MBI. 1 = Warm Acupuncture+Abdominal Needle+Rehabilitation Treatment; 4 = Warm Acupuncture+Rehabilitation Treatment; 5 = Warm Acupuncture+Scalp Needle+Rehabilitation Treatment; 6 = Scalp Needle+Electroacupuncture+Rehabilitation Treatment; 7 = Body Acupuncture+Rehabilitation Treatment; 8 = Electroacupuncture+Rehabilitation Treatment; 10 = Scalp Needle+Body Acupuncture+Rehabilitation Treatment; 12 = acupotome+Rehabilitation Treatment; 14 = Abdominal Needle+Electroacupuncture+Body Acupuncture+Rehabilitation Treatment; 15 = Body Acupuncture; 16 = Rehabilitation Treatment; 17 = Scalp Needle+Wrist Ankle Needle+Rehabilitation Treatment.

**Figure 12:**
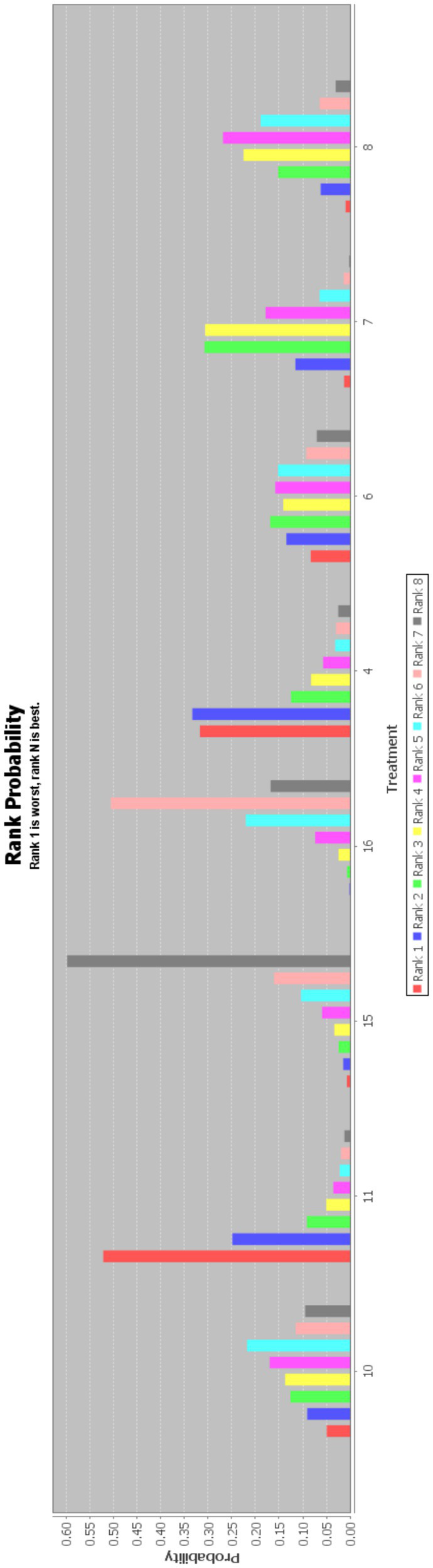
Ranking probability figure for MAS. 4 = Warm Acupuncture+Rehabilitation Treatment; 6 = Scalp Needle+Electroacupuncture+Rehabilitation Treatment; 7 = Body Acupuncture+Rehabilitation Treatment; 8 = Electroacupuncture+Rehabilitation Treatment; 10 = Scalp Needle+Body Acupuncture+Rehabilitation Treatment; 11 = Fire Acupuncture+Body Acupuncture+Rehabilitation Treatment; 15 = Body Acupuncture; 16 = Rehabilitation Treatment.

**Figure 13:**
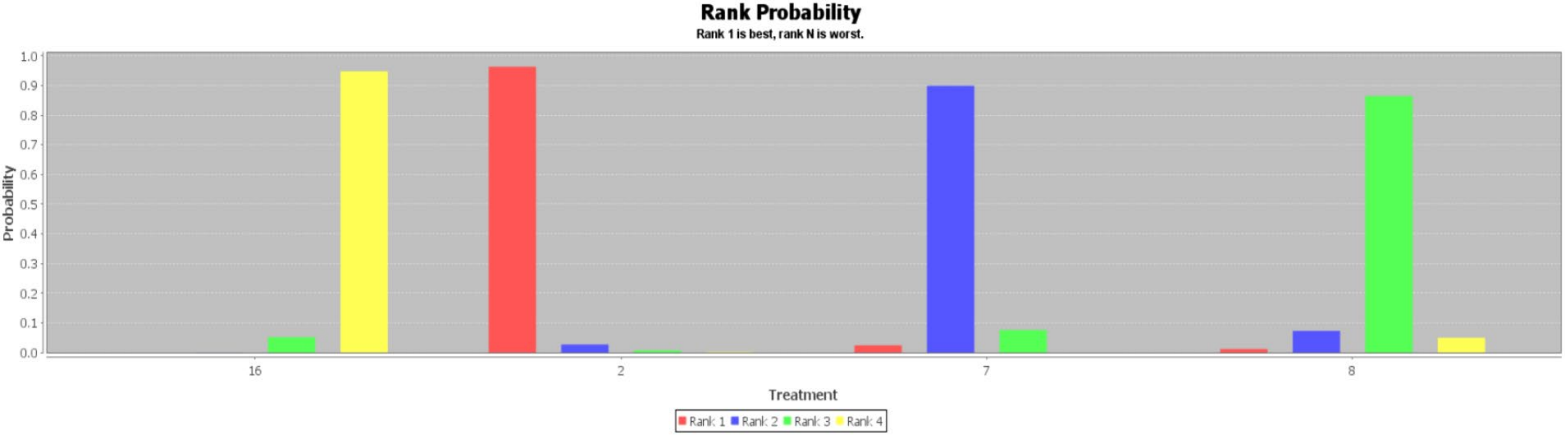
Ranking probability figure for BBS. 2 = Electroacupuncture+Body Acupuncture+Rehabilitation Treatment; 7 = Body Acupuncture+Rehabilitation Treatment; 8 = Electroacupuncture+Rehabilitation Treatment; 16 = Rehabilitation Treatment.

In this paper, the consensus model is adopted and ADDIS is used to rank the various interventions in NMA and the results are represented by a ranking probability matrix (Figures 10–13). The ranking value corresponding to each intervention represents its probability. As can be seen from Figure 10, SN + BA+RT(3) may have the best improvement effect on FMA-LE, followed by EA + BA+RT(2); Figure 11 shows that WA + RT(4) and AN+EA + BA+RT(14) were the most effective interventions in terms of improving MBI scores; Figure 12 shows that FA + BA+RT(11) and WA + RT(4) are the best choices in terms of MAS reduction; Figure 13 shows that in improving BBS, EA + BA+RT(2) is relatively the most effective acupuncture treatment.

### Sensitivity analysis

Sensitivity analyses were performed on all pairwise meta-analyses after excluding trials with high risk of bias. The results show that except for one intervention comparison (BA+RT vs. RT) with BBS as the outcome index was changed (SMD1.62, 95%CI-0.06 to 3.29), no other intervention comparison results were significantly changed.

### Publication bias

**Figure 14:**
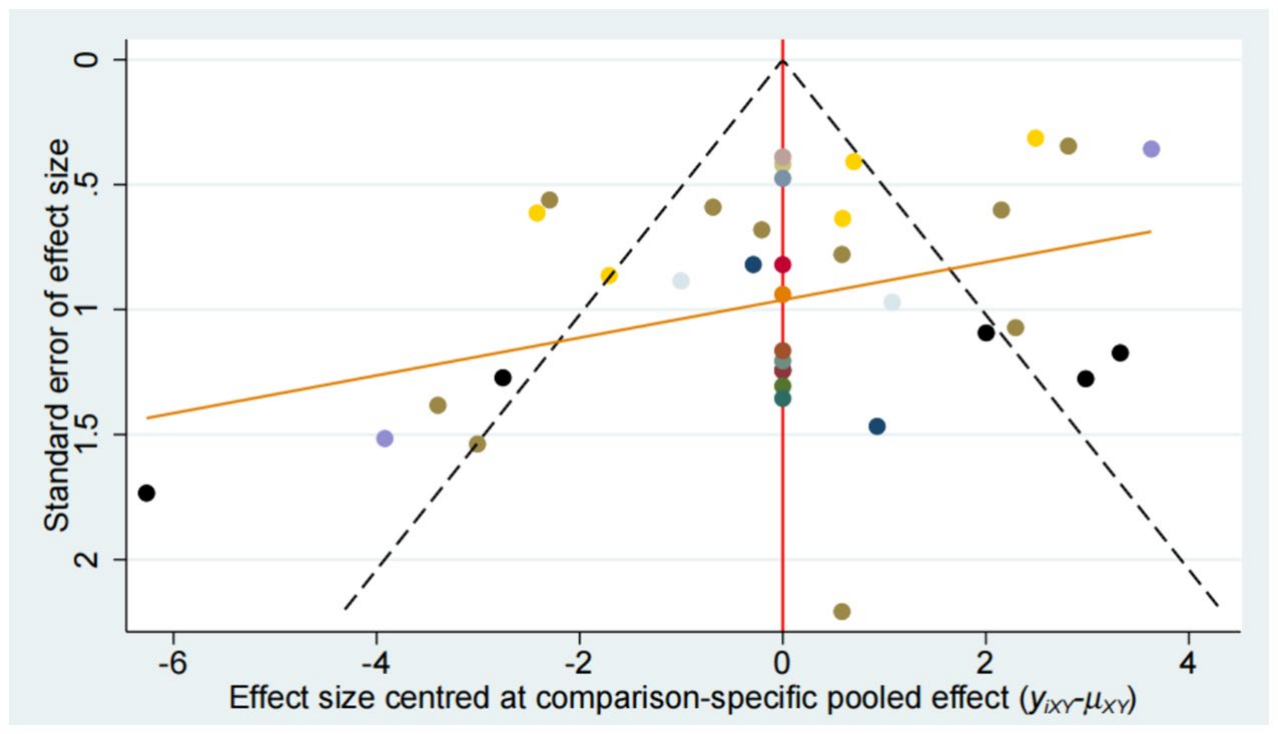
Funnel plot for the network meta-analysis of FMA-LE.

**Figure 15:**
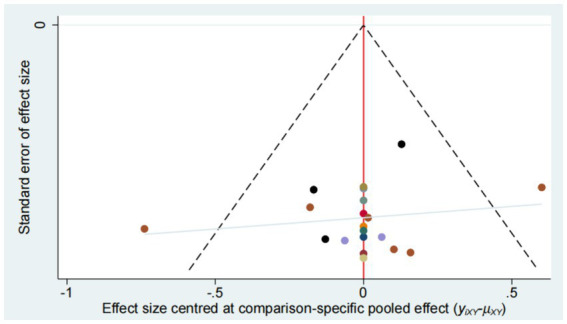
Funnel plot for the network meta-analysis of MBI.

**Figure 16:**
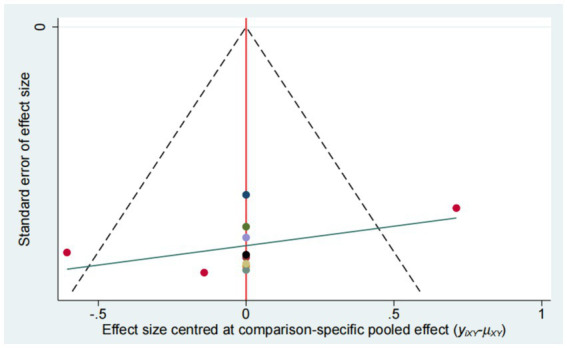
Funnel plot for the network meta-analysis of MAS.

**Figure 17:**
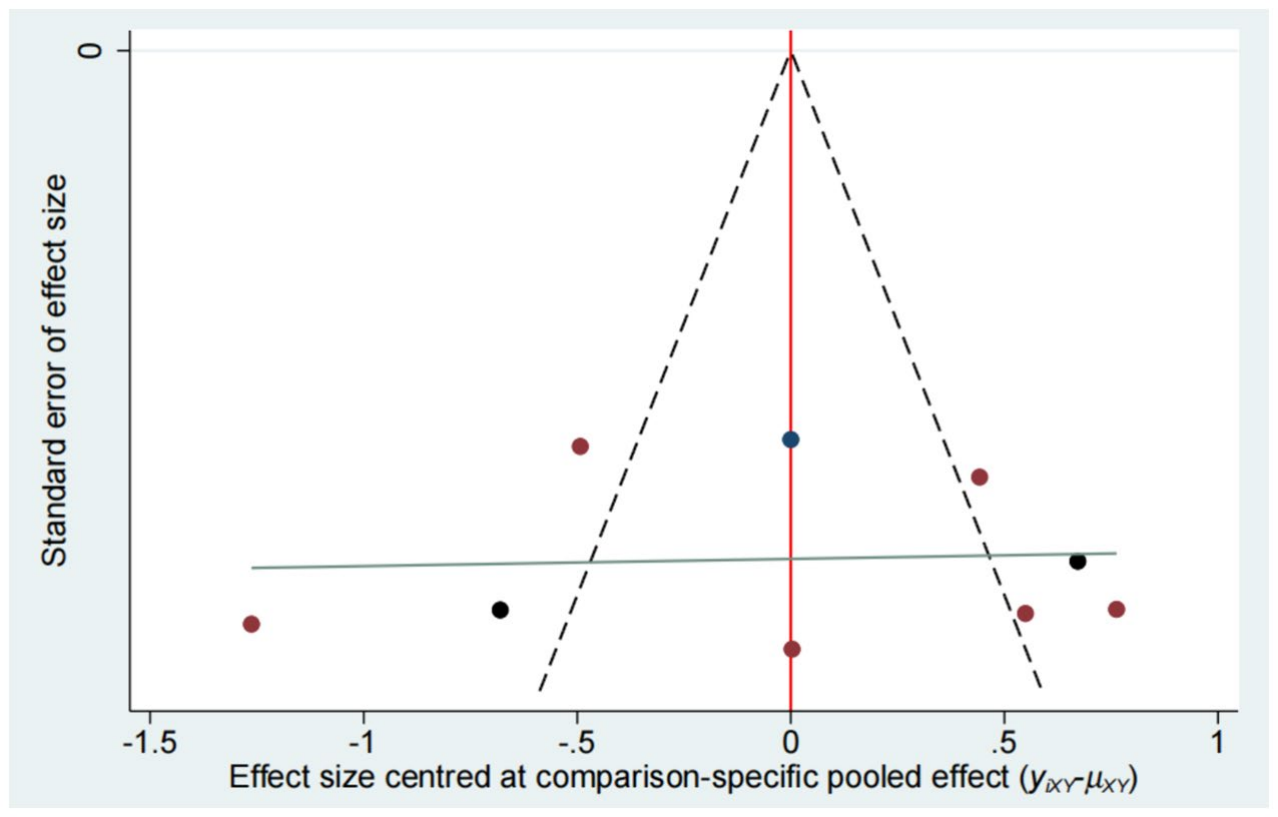
Funnel plot for the network meta-analysis of BBS.

Publication bias was evaluated using funnel plot (Figures 14–17). The results in the figure show that the number of trials involved in the four outcome indicators is relatively symmetrical, with most points evenly distributed on both sides of the middle line and concentrated in the middle area. Therefore, we believe that most of the included studies have a low degree of bias. However, some points were outside the 95% confidence interval, indicating potential heterogeneity in some experiments.

### Adverse events

In this study, adverse events were recorded in 3 RCTS (53, 58, 59), with a total of 17 adverse events. In one of the trials (BA+SN + RT vs. RT) (53), 1 patient had pneumonia, 1 patient had heart disease, and 1 patient had a second stroke. In one trial (SN + EA + RT vs. RT) (58), 2 patients developed subcutaneous hematoma after acupuncture. In one trial (EA + RT vs. RT) (59) 12 people had a second stroke.

## Discussion

### Summary of the main results

Acupuncture has certain advantages in the treatment of lower limb motor dysfunction after stroke (62). Existing studies have shown that acupuncture can reduce lower limb spasm, improve balance function and restore muscle strength, etc. (63, 64) Therefore, acupuncture has been widely used in clinical practice, and is recommended by the World Health Organization as a complementary alternative therapy for post-stroke treatment and care (65). However, there are various ways of acupuncture, and all kinds of the differences of curative effect between acupuncture method is unclear. Therefore, we tried to conduct a comparative study of various acupuncture methods by means of NMA. We conducted a systematic review and Bayesian network meta-analysis on the relevant evidence of 43 RCTs with a large population of patients (*n* = 4,134). In this paper, a variety of clinical acupuncture related therapies for post-stroke limb dysfunction were selected for research, including body acupuncture, electroacupuncture, scalp needle, abdominal needle, warm acupuncture, fire acupuncture, eyes Acupuncture, acupotome and wrist ankle needle.

In pairwise meta-analysis, the proportion of RCTS involving BA+RT compared with other intervention methods was the largest, and when it was compared with RT, which was the most common control group, it was found that this scheme was the best choice among all outcome indicators, and it also involved the largest number of literatures and high quality of evidence. Therefore, we believe that BA+RT may be one of the best acupuncture-related treatment options to promote the recovery of lower limb motor function. However, the results also found that BA+RT was not dominant in comparison with other acupuncture methods combined with RT under each outcome index. Nonetheless, due to the small number of articles related to intervention and insufficient quality of evidence, we cannot definitively conclude that other acupuncture interventions are superior to body acupuncture. Therefore, high-quality RCTs based on different acupuncture methods are still needed to explore the best acupuncture intervention plan in the clinical application of acupuncture to promote the recovery of lower limb function after stroke. In addition, a few acupuncture-related interventions showed different results in different outcome indicators, for example, SN + EA + RT vs. RT showed no significant difference in FMA-LE, but there were differences in MBI and MAS, indicating that the focus of this acupuncture method may be skewed toward the relief of limb spasm and the improvement of overall quality of life.

According to the NMA results, all of the treatments have a positive effect on the recovery of lower limb motor function after stroke, but the difference between most acupuncture treatments is not significant. However, half of the treatments were superior to the two simple interventions of BA and RT, indicating that the clinical efficacy of most rehabilitation programs involving acupuncture was superior to acupuncture alone or conventional rehabilitation treatment alone. Therefore, multiple acupuncture plus routine rehabilitation may have positive significance for promoting post-stroke clinical recovery. In addition, the optimal acupuncture regimen under different outcome indexes was not consistent. In terms of FMA-LE, EyA + EA + BA+RT ranked the highest, and WA + RT was the best choice in terms of MBI. For MAS and BBS, there are FA + BA+RT and EA + BA+RT, respectively. These results indicate that different acupuncture regiments may have different effects on different aspects of limb function recovery after stroke.

The best treatment for FMA-LE and BBS were both included EA + BA+RT, which indirectly confirmed the effectiveness of this intervention, and also indicated that it may have the best effect in promoting the recovery of limb motor function and relieving spasticity. Studies have found that the use of EA in the acute stage of infarction can effectively improve the collateral circulation in the central area, increase cerebral blood perfusion, and reshape the brain motor function network (66, 67). For the peripheral aspects, electroacupuncture can reduce the excitation threshold of nerve and muscle cells, increase the release of endogenous morphine peptides, so as to stimulate the antagonistic muscles of the affected limb, inhibit the muscle strength of the spastic muscles, and relieve the spasm of the limb (68). To sum up, EA may have the best effect on the improvement of limb movement and spasticity. MBI shows that WA + RT has the best curative effect. Relevant studies (69, 70) have found that warm acupuncture can enhance the blood supply of nerve cells, promote axon regeneration to promote the overall recovery of the central nervous system on the one hand, and promote the reconstruction of reflex arc and prevent muscle atrophy on the other hand, so as to improve muscle tension, relieve spasm, and further promote the recovery of local lesions. The above evidence indicates that acupuncture plus moxibustion may not only have positive significance on the recovery of limb function, but also improve the overall quality of daily life of patients to a certain extent. MAS showed that FA + BA+RT had better effect, which may suggest that the fire needle has certain advantages in relieving limb spasm. Relevant studies (71, 72) have shown that the anti-spasticity mechanism of fire needle is mainly related to neurotransmitters and receptors related to spasticity, and it can relieve spasticity by increasing the expression of inhibitory transmitters or reducing the expression of excitatory neurotransmitters. Meanwhile, a meta-analysis (73) has shown that fire needle is better than ordinary acupuncture in relieving limb spasticity after stroke. However, there was only one fireneedle-related RCTs included in the MAS evaluation, so there may be some bias.

The above evidence indicates that BA+RT may currently be the preferred clinical regimen for most acupuncture treatment of lower limb motor dysfunction after stroke. On this basis, the regimen consisting of other acupuncture methods, such as electroacupuncture, warm acupuncture and fire acupuncture, may produce more optimistic therapeutic effects for patients with different prognostic priorities.

### Overall quality of evidence

All RCTS included in this study were rigorously screened. In terms of adverse events, a total of 17 adverse events were recorded in three RCTs (53, 58, 59). Among them, secondary stroke accounts for a relatively large proportion, and almost all of them come from one rct (59) (12/13). On the one hand, it may be due to the large sample size of this study, and on the other hand, it may be due to the high incidence of secondary stroke itself. According to statistics (74), about 1/4 of stroke patients in the United States will have secondary stroke. At the same time, some studies suggest that the second stroke may initiate the brain’s self-protection and potential repair function (75). Therefore, the results do not indicate that there is an inevitable relationship between acupuncture and the occurrence of secondary stroke.

It is difficult to achieve blindness in clinical trials of acupuncture. First of all, it is impractical to blind acupuncture operators, and it is difficult to blind patients in the comparison between acupuncture and conventional rehabilitation. Therefore, in the process of bias risk assessment, all RCTs performance bias compared with non-acupuncture were labeled as high risk. For these reasons, we conducted a sensitivity analysis of the included studies and found that most of the results were reliable. In addition, we also pay attention to publication bias. For the four outcome measures, a small number of RCTs were outside the 95% confidence interval, suggesting some potential heterogeneity.

### Strengths and limitations

The strengths of this study are as follows. First, the study was reviewed in strict accordance with the PRISMA-NMA (16) and PRISMA guidelines and checklists (76). Second, to ensure the adequacy of the number of references, 8 Chinese and English databases and 2 clinical trial registries were searched, and the search results only included RCTS. Third, the Bayesian analysis method adopted in this study is more advantageous than the frequency-based method, and at the same time, the ADDIS software we used has been recognized by some statisticians (77). Fourth, this is the first study to evaluate the efficacy and safety of different acupuncture-related treatments for Stroke. we evaluated up to 16 different modalities of acupuncture combined with RT, and screened the relatively best acupuncture intervention under different outcome indicators, in order to assist clinicians in selecting the most optimal acupuncture treatment for different patients.

This study also has some limitations. First of all, the data included in this study were the results of the baseline period and the first time after the end of treatment, and the long-term follow-up data after acupuncture treatment was not included in the analysis. Differences in the course of disease, duration of treatment, and duration of treatment among different studies may increase the risk of bias in study results. We documented patient gender, acupuncture point selection, treatment frequency, and other factors that may have contributed to heterogeneity in the article, but did not include these factors in the analysis, which may also have contributed to some heterogeneity in the results. Some acupuncture methods with relatively few clinical applications, such as abdominal acupuncture, acupotomy, ear acupuncture, etc., were included in the inclusion and analysis process, due to the insufficient record of outcome indicators in some literatures, the number of relevant literatures included in this part of acupuncture methods was very small, which may have a certain impact on the final results. At the same time, since most of the literatures are from China and only one is from the United States, there may be some heterogeneity in the analysis results. In addition, ischemic stroke and hemorrhagic stroke were included in this study, but the two diseases were not discussed separately, but were combined for analysis, which may also affect the accuracy of the results.

## Conclusion

This study provides sufficient evidence to prove that acupuncture combined with rehabilitation is superior to rehabilitation alone in the treatment of lower limb motor dysfunction after stroke. BA+RT is an acupuncture-related treatment with the most clinical use, the highest quality of evidence and the best efficacy compared with rehabilitation. However, due to the limited number and quality of the included literature, we recommend that more high-quality, large-sample, multicenter randomized controlled clinical trials be conducted to further validate these results.

## Data availability statement

The datasets presented in this study can be found in online repositories. The names of the repository/repositories and accession number(s) can be found in the article/Supplementary material.

## Author contributions

YL: Data curation, Investigation, Writing – original draft, Writing – review & editing. YT: Conceptualization, Methodology, Writing – original draft. LW: Software, Visualization, Writing – original draft. PY: Data curation, Supervision, Writing – original draft. CW: Investigation, Project administration, Writing – original draft. LZe: Resources, Validation, Writing – review & editing. JY: Methodology, Supervision, Writing – review & editing. LZh: Funding acquisition, Writing – review & editing.
