# Supplementary material for: Optimal acupuncture methods for lower limb motor dysfunction after stroke: a systematic review and network meta-analysis
Source: Front Neurol. 2024 Jul 10;15:1415792. doi: 10.3389/fneur.2024.1415792 (PMC11270540; doi:10.3389/fneur.2024.1415792)
Supplement: Supplementary file 1 [file Data_Sheet_1.pdf]

**Additional file 1.** The PRISMA network meta-analysis checklist.

**Additional file 2.** Main characteristics of included RCTs.

**Additional file 3.** Risk of Bias Assessment.

**Additional file 4.** Pairwise Meta-Analysis.

**Additional file 5.** Assessment of inconsistency results.

**Additional file 6.** Assessment of convergence of the model results.

**Additional file 1.** The PRISMA network meta-analysis checklist.

Notes: From *Annals of Internal Medicine*, Brian Hutton, Georgia Salanti, Deborah M. Caldwell, et al , The PRISMA Extension Statement for Reporting of Systematic Reviews Incorporating Network Meta-analyses of Health Care Interventions: Checklist and Explanations, 2015;162(11):777–784.
Copyright © 2015 American College of Physicians. All Rights Reserved. Reprinted with the permission of American College of Physicians, Inc.[24]

| Section/Topic | Item # | Checklist Item |
|---|---|---|
| **TITLE** | | |
| Title | 1 | Identify the report as a systematic review *incorporating a network meta-analysis (or related form of meta-analysis).* |
| **ABSTRACT** | | |
| Structured summary | 2 | Provide a structured summary including, as applicable:<br>**Background:** main objectives<br>**Methods:** data sources; study eligibility criteria, participants, and interventions; study appraisal; and *synthesis methods, such as network meta-analysis.*<br>**Results:** number of studies and participants identified; summary estimates with corresponding confidence/credible intervals; *treatment rankings may also be discussed. Authors may choose to summarize pairwise comparisons against a chosen treatment included in their analyses for brevity.*<br>**Discussion/Conclusions:** limitations; conclusions and implications of findings.<br>**Other:** primary source of funding; systematic review registration number with registry name. |
| **INTRODUCTION** | | |
| Rationale | 3 | Describe the rationale for the review in the context of what is already known*, including mention of why a network meta-analysis has been conducted.*_ |
| Objectives | 4 | Provide an explicit statement of questions being addressed, with reference to participants, interventions, comparisons, outcomes, and study design (PICOS). |
| **METHODS** | | |
| Protocol and registration | 5 | Indicate whether a review protocol exists and if and where it can be accessed (e.g., Web address); and, if available, provide registration information, including registration number. |

| | | |
|---|---|---|
| Eligibility criteria | 6 | Specify study characteristics (e.g., PICOS, length of follow-up) and report characteristics (e.g., years considered, language, publication status) used as criteria for eligibility, giving rationale. *Clearly describe eligible treatments included in the treatment network, and note whether any have been clustered or merged into the same node (with justification).*_ |
| Information sources | 7 | Describe all information sources (e.g., databases with dates of coverage, contact with study authors to identify Additional file studies) in the search and date last searched. |
| Search | 8 | Present full electronic search strategy for at least one database, including any limits used, such that it could be repeated. |
| Study selection | 9 | State the process for selecting studies (i.e., screening, eligibility, included in systematic review, and, if applicable, included in the meta-analysis). |
| Data collection process | 10 | Describe method of data extraction from reports (e.g., piloted forms, independently, in duplicate) and any processes for obtaining and confirming data from investigators. |
| Data items | 11 | List and define all variables for which data were sought (e.g., PICOS, funding sources) and any assumptions and simplifications made. |
| **Geometry of the network** | **S1** | Describe methods used to explore the geometry of the treatment network under study and potential biases related to it. This should include how the evidence base has been graphically summarized for presentation, and what characteristics were compiled and used to describe the evidence base to readers. |
| Risk of bias within individual studies | 12 | Describe methods used for assessing risk of bias of individual studies (including specification of whether this was done at the study or outcome level), and how this information is to be used in any data synthesis. |
| Summary measures | 13 | State the principal summary measures (e.g., risk ratio, difference in means). *Also describe the use of Additional file summary measures assessed, such as treatment rankings and surface under the cumulative ranking curve (SUCRA) values, as well as modified approaches used to present summary findings from meta-analyses.* |
| Planned methods of analysis | 14 | Describe the methods of handling data and combining results of studies for each network meta-analysis. This should include, but not be limited to:<br>• *Handling of multi-arm trials;*<br>• *Selection of variance structure;*<br>• *Selection of prior distributions in Bayesian analyses; and* |

| | | |
|---|---|---|
| | | • *Assessment of model fit.* |
| **Assessment of Inconsistency** | **S2** | Describe the statistical methods used to evaluate the agreement of direct and indirect evidence in the treatment network(s) studied. Describe efforts taken to address its presence when found. |
| Risk of bias across studies | 15 | Specify any assessment of risk of bias that may affect the cumulative evidence (e.g., publication bias, selective reporting within studies). |
| Additional file analyses | 16 | Describe methods of Additional file analyses if done, indicating which were pre-specified. This may include, but not be limited to, the following:<br>• Sensitivity or subgroup analyses;<br>• Meta-regression analyses;<br>• *Alternative formulations of the treatment network; and*<br>• *Use of alternative prior distributions for Bayesian analyses (if applicable).*_ |

**RESULTS†**

| | | |
|---|---|---|
| Study selection | 17 | Give numbers of studies screened, assessed for eligibility, and included in the review, with reasons for exclusions at each stage, ideally with a flow diagram. |
| **Presentation of network structure** | **S3** | Provide a network graph of the included studies to enable visualization of the geometry of the treatment network. |
| **Summary of network geometry** | **S4** | Provide a brief overview of characteristics of the treatment network. This may include commentary on the abundance of trials and randomized patients for the different interventions and pairwise comparisons in the network, gaps of evidence in the treatment network, and potential biases reflected by the network structure. |
| Study characteristics | 18 | For each study, present characteristics for which data were extracted (e.g., study size, PICOS, follow-up period) and provide the citations. |
| Risk of bias within studies | 19 | Present data on risk of bias of each study and, if available, any outcome level assessment. |
| Results of individual studies | 20 | For all outcomes considered (benefits or harms), present, for each study: 1) simple summary data for each intervention group, and 2) effect estimates and confidence intervals. *Modified approaches may be needed to deal with information from larger networks.* |

| | | |
|---|---|---|
| Synthesis of results | 21 | Present results of each meta-analysis done, including confidence/credible intervals. *In larger networks, authors may focus on comparisons versus a particular comparator (e.g. placebo or standard care), with full findings presented in an appendix. League tables and forest plots may be considered to summarize pairwise comparisons.* If Additional file summary measures were explored (such as treatment rankings), these should also be presented. |
| **Exploration for inconsistency** | **S5** | Describe results from investigations of inconsistency. This may include such information as measures of model fit to compare consistency and inconsistency models, *P* values from statistical tests, or summary of inconsistency estimates from different parts of the treatment network. |
| Risk of bias across studies | 22 | Present results of any assessment of risk of bias across studies for the evidence base being studied. |
| Results of Additional file analyses | 23 | Give results of Additional file analyses, if done (e.g., sensitivity or subgroup analyses, meta-regression analyses*, alternative network geometries studied, alternative choice of prior distributions for Bayesian analyses,* and so forth). |
| **DISCUSSION** | | |
| Summary of evidence | 24 | Summarize the main findings, including the strength of evidence for each main outcome; consider their relevance to key groups (e.g., healthcare providers, users, and policy-makers). |
| Limitations | 25 | Discuss limitations at study and outcome level (e.g., risk of bias), and at review level (e.g., incomplete retrieval of identified research, reporting bias). *Comment on the validity of the assumptions, such as transitivity and consistency. Comment on any concerns regarding network geometry (e.g., avoidance of certain comparisons).* |
| Conclusions | 26 | Provide a general interpretation of the results in the context of other evidence, and implications for future research. |
| **FUNDING** | | |
| Funding | 27 | Describe sources of funding for the systematic review and other support (e.g., supply of data); role of funders for the systematic review. This should also include information regarding whether funding has been received from manufacturers of treatments in the network and/or whether some of the authors are content experts with professional conflicts of interest that could affect use of treatments in the network. |

**Additional file 2.** Main characteristics of included RCTs.

| Author year | Nation | Types of stroke(CH:Cerebral Infarction;CI:Cerebral Hemorrhage) | Stage of stroke | Brunnstrom stage | Course of disease（D） Treatment group | Control group 1 | Control group 2 | Sample size (Shedding number) | Sample ratio | Age | Gender (M:F) | (A) Treatment group | (B) Control group 1 | (C) Control group 2 | Duration of treatment(d) | Outcome index | Result |
|---|---|---|---|---|---|---|---|---|---|---|---|---|---|---|---|---|---|
| Wang Y 2022 | CN | - | | - | 26. 01±3. 83 | 25. 63 ±3. 12 | | 120 | 1：1 | A:69.89±2. 04;B:69.63 ±2.11 | A:(33:27);B :(32:28) | WA+AN+R T | RT | | 28 | 1.FMA;2.M AS;3.MBI; | 1.A＞B;2.A＞ B;3.A＞B |
| Wu L 2021 | CN | A:(CH:CI=32:35);B:(CH:C I=28:39) | | II～IV | 73.25±14.98 | 69.03±1 5.17 | | 134 | 1：1 | A:57.63±7. 51;B:58.43 ±7.29 | A:(30:26);B :(28:28) | EA+BA+R T | BA+RT | | 180 | 1.FMA;2.B BS | 1.A＞B;2.A＞B |
| Zeng Y 2021 | CN | - | | II~IV | 36.80±13.35 | 36.50±9. 69 | | 112 | 1：1 | A:69.23±9. 43;B:67.91 ±12.34 | A:(35:32);B :(40:27) | EyA+EA+B A+RT | EA+BA+R T | | | 1.FMA | 1.A=B |
| Bo F 2021 | CN | A:(CH:CI=7:24);B:(CH:CI =9:21) | | - | - | - | | 64(3) | 1：1 | A:59.91±1 2.41;B:56. 48±10.30 | A:(18:13);B :(22:8) | BA+RT | RT | | 28 | 1.BBS | 1.A＞B |
| You M 2021 | CN | A:(CH:CI=7:24);B:(CH:CI =9:21) | | II～IV | - | - | | 53 | 1：1 | A:62. 63±1 4. 49;B:66 . 38±12. 06 | A:(21:6);B:( 15:11) | SN+WAN+ RT | RT | | 20 | 1.MAS;2.M BI | 1.A＞B;2.A＞B |

| | | | | | | | | | | | | | | | |
|---|---|---|---|---|---|---|---|---|---|---|---|---|---|---|---|
| Yin G 2021 | CN | CI | | - | - | - | 72 | 1：1 | A:59.24±5.71;B:58.83±5.46 | A:(21:15);B:(19:17) | WA+RT | RT | 28 | 1.FMA;2.MBI | 1.A＞B;2.A＞B |
| Huang Z 2018 | CN | A:(CH:CI=23:17);B:(CH:CI=21:19) | | - | 34.22±12.10 | 37.91±11.62 | 80 | 1：1 | A:65.39±7.34;B:67.79±9.84 | A:(22:18);B:(24:16) | WA+SN+RT | RT | 28 | 1.FMA;2.MBI | 1.A＞B;2.A＞B |
| Zhang L 2021 | CN | A:(CH:CI=10:20);B:(CH:CI=11:19) | | - | 35. 65±10. 31 | 37．20 ± 7．88 | 60 | 1：1 | A:62. 14±8. 03;B:59. 36±6．95 | A:(17:13);B:(16:14) | SN+EA+RT | RT | 28 | 1.FMA;2.MBI；3.MAS | 1.A＞B;2.A＞B;3.A＞B |
| Duan Y 2021 | CN | A:(CH:CI=13:33);B:(CH:CI=21:26) | | I～IV | 36.85±37.62 | 37.44±61.95 | 103 | 1：1 | A:59.15±9.78;B:59.63±9.86 | A:(27:19);B:(33:24) | NN+RT | EA+RT | 20 | 1.MBI;2.MAS | 1.A=B;2.A＞B |
| Zhu G 2020 | CN | A:(CH:CI=11:24);B:(CH:CI=13:22) | | - | - | - | 70 | 1：1 | A:61. 43±8. 35;B:60. 71±8．13 | A:(21:14);B:(20:15) | BA+RT | RT | 42 | 1.FMA | 1.A＞B |
| Hao Q 2020 | CN | A:(CH:CI=23:13);B:(CH:CI=20:16) | | II～IV | 120.01±0.43 | 120.56±0.51 | 72 | 1：1 | A:56.16±9.70;B:54.48±8.50 | A:(25:11);B:(27:9) | EA+RT | RT | 28 | 1.FMA;2.MBI;3.MAS;4.BBS | 1.A＞B;2.A＞B;3.A＞B;4.A＞B |
| Li D 2020 | CN | A:(CH:CI=6:35);B:(CH:CI=3:38) | | II～III | 29.48±6.32 | 28.71±5.38 | 82 | 1：1 | A:56.16±9.70;B:54.48±8.50 | A:(25:16);B:(28:13) | FA+RT | EA+RT | 56 | 1.FMA;2.MAS | 1.A＞B;2.A=B |
| Sun S 2020 | CN | CI | Acute stage（less than 24h） | - | - | - | 108 | 1：1 | A:59.69;B:60.11 | A:(25:29);B:(24:30) | BA+RT | RT | 28 | 1.MAS;2.MBI;3.BBS | 1.A＞B;2.A＞B;3.A＞B |

| | | | | | | | | | | | | | | | | |
|---|---|---|---|---|---|---|---|---|---|---|---|---|---|---|---|---|
| Liu C 2020 | CN | A:(CH:CI=14:16);B:(CH:CI=12:18) | II~V | 94.40±38.45 | 92.36±35.31 | | 60 | 1：1 | A:61.72±5.82;B:60.53±6.73 | A:(15:15);B:(14:16) | FA+BA+RT | BA+RT | | 14 | 1.MAS;2.FMA | 1.A＞B;2.A＞B |
| Cui J 2020 | CN | - | - | 25.87±7.13 | 25.65±7.01 | | 70 | 1：1 | A:58.78±10.53;B:58.63±10.65 | A:(19:16);B:(20:15) | WA+RT | RT | | 28 | 1.FMA | 1.A＞B |
| Dang H 2019 | CN | CI | - | 21.85±2.27 | 22.09±2.87 | | 100 | 1：1 | A:52.45±4.06;B:52.28±3.29 | A:(26:24);B:(27:23) | BA+RT | RT | | 30 | 1.FMA;2.BBS;3.MBI | 1.A＞B;2.A＞B;3.A＞B |
| Wang H 2019 | CN | CH | III～IV | 59.53±17.49 | 55.72±15.78 | | 59 | 1：1 | A:56.7±7.02;B:59±7.51 | A:(19:11);B:(16:13) | SN+BA+RT | RT | | 28 | 1.FMA;2.MBI；3.MAS | 1.A＞B;2.A=B;3.A＞B |
| Wang Y 2019 | CN | A:(CH:CI=17:39);B:(CH:CI=18:38);C(CH:CI=18:38) | - | 11.4±5.4 | 11.9±4.6 | 11.6±5.2 | 168 | 1:1:1 | A:64.5±9.6;B:64.8±9.3;C:64.3±9.7 | A:(29:27);B:(29:27);C:(28:28) | BA+RT | BA | RT | 42 | 1.FMA | 1.A＞B=C |
| Chen J 2018 | CN | A:(CH:CI=30:22);B:(CH:CI=27:25) | - | 53.2±14.4 | 51.5±16.5 | | 104 | 1：1 | A:58.4±7.8;B:57.1±7.2 | A:(35:17);B:(33:19) | BA+RT | RT | | 42 | 1.FMA;2.MBI | 1.A＞B;2.A＞B |
| Zhao M 2018 | CN | A:(CH:CI=15:15);B:(CH:CI=16:14) | - | 87.87±38.31 | 90.37±42.71 | | 64（4） | 1：1 | A:62.47±10.01;B:59.90±12.39 | A:(17:13);B:(19:11) | FA+RT | BA+RT | | 42 | 1.FMA;2.MAS | 1.A＞B;2.A＞B |

| | | | | | | | | | | | | | | | | |
|---|---|---|---|---|---|---|---|---|---|---|---|---|---|---|---|---|
| Hou X 2018 | CN | - | - | 3.24±0.56 | 3.35±0.67 | | 96 | 1：1 | A:58.37±5.67;B:59.19±6.13 | A:(34:14);B:(35:13) | FA+RT | RT | | 28 | 1.FMA | 1.A＞B |
| Tan S 2018 | CN | A:(CH:CI=25:19);B:(CH:CI=21:23) | ≥II | 44.86±6.34 | 44.62±6.28 | | 88 | 1：1 | A:54.93±7.82;B:54.78±7.69 | A:(22:22);B:(24:20) | BA+RT | RT | | 84 | 1.FMA;2.MBI；3.MAS | 1.A＞B;2.A＞B;3.A＞B |
| Xu Y 2018 | CN | A:(CH:CI=25:19);B:(CH:CI=21:23) | III～V | 90.30±39.9 | 105.6±50.4 | | 84 | 1：1 | A:48. 59±13．45;B:49. 27±14. 56 | A:(22:19);B:(23:20) | EA+RT | RT | | 42 | 1.FMA;2.BBS | 1.A＞B;2.A＞B |
| Wang X 2017 | CN | CI | - | 93.6±44.4 | 103.2±47.7 | | 78 | 1：1 | A:56. 61±14. 36;B:55. 33±15. 49 | A:(20:19);B:(21:18) | EA+RT | BA+RT | | 24 | 1.FMA;2.MAS | 1.A＞B;2.A＞B |
| Song Y 2017 | CN | A:(CH:CI=33:47);B:(CH:CI=29:51) | - | 43.8±16.2 | 41.4±15.3 | | 160 | 1：1 | A:57.45±8.14;B:56.71±7.63 | A:(55:25);B:(52:28) | BA+RT | RT | | 30 | 1.FMA;2.BBS | 1.A＞B;2.A＞B |
| Xu L 2017 | CN | - | III～IV | 37.6±10.6 | 33.7±4.5 | 38.4±10.2 | 90 | 1:1:1 | A:58±10;B:55±5;C:61±10 | A:(15:15);B:(17:13);C:(16:14) | BA+RT | BA | RT | 40 | 1.FMA;2.MBI；3.MAS | 1.A＞B=C;2.A＞B=C;3.A＞B=C |
| Yang R 2017 | CN | A:(CH:CI=13:17);B:(CH:CI=15:15) | - | 22.10±2.09 | 22.00±2.98 | | 60 | 1：1 | A:71±3;B:70±3 | A:(17:13);B:(15:15) | NN+RT | RT | | 28 | 1.FMA | 1.A＞B |

| | | | | | | | | | | | | | | | |
|---|---|---|---|---|---|---|---|---|---|---|---|---|---|---|---|
| **Chen D 2017** | **CN** | **A:(CH:CI=11:37);B:(CH:CI=10:38)** | | **-** | **41.13±32.82** | **40.92±28.19** | **100（4）** | **1：1** | **A:67±11;B:67±11** | **A:(29:19);B:(30:18)** | **EA+BA+SN+RT** | **RT** | **28** | **1.FMA** | **1.A＞B** |
| **Zeng Y 2016** | **CN** | **A:(CH:CI=11:39);B:(CH:CI=14:36)** | **Subacute stage** | **-** | **34.74±2.92** | **34.18±2.86** | **100** | **1：1** | **A:66±12;B:68±10** | **A:(30:20);B:(32:18)** | **SN+BA+RT** | **RT** | **56** | **1.FMA;2.MBI** | **1.A＞B;2.A＞B** |
| **Xu S 2016** | **CN** | **-** | | **III～V** | **50.39±22.52** | **47.75±22.63** | **76(5)** | **1：1** | **A:60±10;B:65±6** | **A:(28:8);B:(24:11)** | **SN+BA+RT** | **RT** | **28** | **1.FMA** | **1.A＞B** |
| **Qi Y 2016** | **CN** | **A:(CH:CI=27:22);B:(CH:CI=29:20)** | | **-** | **38.2±13.9** | **34.7±11.6** | **98** | **1：1** | **A:67±7;B:65±8** | **A:(29:20);B:(25:24)** | **BA+RT** | **RT** | **42** | **1.FMA;2.MAS** | **1.A＞B;2.A＞B** |
| **Li Z 2016** | **CN** | **A:(CH:CI=27:22);B:(CH:CI=29:20)** | | **-** | **4.56±1.67** | **3.96±2.05** | **60** | **1：1** | **A:65±7.92;B:59±8.45** | **A:(20:10);B:(17:13)** | **SN+BA+RT** | **RT** | **21** | **1.FMA** | **1.A＞B** |
| **Wang N 2015** | **CN** | **A:(CH:CI=6:24);B:(CH:CI=8:22)** | | **-** | **253.4±167.43** | **247.4±171.13** | **60** | **1：1** | **A:54±20;B:53±21** | **A:(18:12);B:(17:13)** | **FA+RT** | **RT** | **30** | **1.FMA;2.MAS** | **1.A＞B;2.A＞B** |
| **Wang Y 2015** | **CN** | **-** | | **-** | **-** | **-** | **120（2）** | **1：1** | **A:63.98±9.67;B:61.48±11.27** | **A:(38:20);B:(42:18)** | **AN+EA+BA+RT** | **BA+RT** | **28** | **1.FMA;2.MBI** | **1.A＞B;2.A＞B** |
| **Gong W 2015** | **CN** | **A:(CH:CI=14:26);B:(CH:CI=11:29)** | | **-** | **33.82±11.38** | **34.71±12.04** | **80** | **1：1** | **A:57.43±6.11;B:56.12±5.97** | **A:(25:15);B:(23:17)** | **EA+RT** | **RT** | **30** | **1.FMA** | **1.A＞B** |

| | | | | | | | | | | | | | | |
|---|---|---|---|---|---|---|---|---|---|---|---|---|---|---|
| Zhu Y 2013 | CN | A:(CH:CI=34:64);B:(CH:CI=30:60) | - | 15.10±7.00 | 14.40±7.80 | 188 | 1：1 | A:57.43±6.11;B:56.12±5.97 | A:(71:27);B:(59:31) | BA+SN+RT | RT | 90 | 1.FMA;2.MBI | 1.A=B;2.A=B |
| Yin Z 2013 | CN | A:(CH:CI=13:18);B:(CH:CI=11:19) | - | 27.5±13.7 | 26.3±14.5 | 61 | 1：1 | A:47.24±13.05;B:46.09±14.25 | A:(22:9);B:(23:7) | BA+RT | RT | 56 | 1.FMA;2.MBI;3.BBS | 1.A＞B;2.A＞B;3.A＞B |
| Tang Z 2012 | CN | CI | - | - | - | 120 | 1：1 | 43-78 | （68：52） | WA+RT | EA+RT | 30 | 1.FMA;2.MBI;3.MAS | 1.A＞B;2.A＞B;3.A＞B |
| He X 2008 | CN | A:(CH:CI=11:19);B:(CH:CI=14:16) | - | - | - | 60 | 1：1 | A:48±12.5;B:49±13.0 | A:(18:12);B:(17:13) | BA+RT | RT | 30 | 1.FMA;2.BBS | 1.A＞B;2.A＞B |
| David N. Alexander 2004 | USA | A:(CH:CI=2:14);B:(CH:CI=2:13) | - | - | - | 32（1） | 1：1 | A:48±12.5;B:49±13.0 | A:(9:7);B:(8:7) | BA+RT | RT | 14 | 1.FMA | 1.A=B |
| Zhang Q 2022 | CN | A:(CH:CI=7:24);B:(CH:CI=4:26) | - | - | - | 72(11) | 1：1 | - | A:(21:10);B:(18:12) | SN+EA+RT | RT | 28 | 1.FMA;2.MBI;3.MAS | 1.A=B;2.A＞B;3.A＞B |
| Yan L 2022 | CN | A:(CH:CI=60:131);B:(CH:CI=67:124) | II～IV | 252±51 | 246±54 | 382 | 1：1 | A:58.1±10.7;B:57.2±10.5 | A:(101:90);B:(98:93) | EA+RT | RT | 56 | 1.FMA | 1.A＞B |
| Dai G 2016 | CN | A:(CH:CI=20:37);B:(CH:CI=23:34) | - | 38.2±10.4 | 36.7±10.1 | 114 | 1：1 | A:62.7±8.6;B:62.9±9.1 | A:(32:25);B:(36:21) | EA+RT | RT | 28 | 1.FMA | 1.A＞B |

**Additional file 3.** Risk of Bias Assessment.

| author year | Random sequence generation (selection bias) | Allocation concealment (selection bias) | Blinding of participants and personnel (performance bias) | Blinding of outcome assessment (detection bias) | Incomplete outcome data (attrition bias) | Selective reporting (reporting bias) | Other bias |
|---|---|---|---|---|---|---|---|
| Wang Y 2022 | L | N | H | N | L | L | H |
| Wu L 2021 | L | N | N | N | L | L | L |
| Zeng Y 2021 | L | N | N | N | L | L | L |
| Bo F 2021 | L | N | H | N | L | L | H |
| You M 2021 | L | N | H | N | L | L | H |
| Yin G 2021 | L | N | H | N | L | L | N |
| Huang Z 2018 | L | N | H | H | L | L | N |
| Zhang L 2021 | L | N | H | N | L | L | N |

| | | | | | | | |
|---|---|---|---|---|---|---|---|
| Duan Y 2021 | L | N | N | N | L | L | N |
| Zhu G 2020 | L | N | H | H | L | L | N |
| Hao Q 2020 | L | N | H | N | L | L | H |
| Li D 2020 | L | N | N | N | L | L | H |
| Sun S 2020 | L | N | H | N | L | L | L |
| Liu C 2020 | L | N | N | N | L | L | L |
| Cui J 2020 | H | L | H | N | L | L | H |
| Dang H 2019 | L | N | H | N | L | L | L |
| Wang H 2019 | L | L | L | N | L | L | L |
| Wang Y 2019 | L | N | N | N | L | L | L |

| | | | | | | | |
|---|---|---|---|---|---|---|---|
| Chen J 2018 | L | H | H | N | L | L | N |
| Zhao M 2018 | L | N | N | N | L | L | N |
| Hou X 2018 | H | N | H | N | L | L | N |
| Tan S 2018 | L | N | H | N | L | L | N |
| Xu Y 2018 | L | N | H | N | L | L | N |
| Wang X 2017 | L | N | N | N | L | L | N |
| Song Y 2017 | L | N | H | N | L | L | H |
| Xu L 2017 | L | N | N | N | L | L | N |
| Yang R 2017 | L | N | H | H | L | L | N |
| Chen D 2017 | L | H | H | N | L | L | N |

| | | | | | | | |
|---|---|---|---|---|---|---|---|
| Zeng Y 2016 | L | N | H | N | L | L | L |
| Xu S 2016 | L | N | H | N | L | L | H |
| Qi Y 2016 | L | N | H | N | L | L | L |
| Li Z 2016 | L | N | H | H | L | L | N |
| Wang N 2015 | L | N | H | N | L | L | H |
| Wang Y 2015 | L | N | N | N | L | L | N |
| Gong W 2015 | L | N | H | N | L | L | N |
| Zhu Y 2013 | L | L | H | L | L | L | N |
| Yin Z 2013 | L | N | H | N | L | L | H |
| Tang Z 2012 | L | N | N | N | L | L | N |

| | | | | | | | |
|---|---|---|---|---|---|---|---|
| He X 2008 | H | N | H | N | L | L | H |
| David N. Alexander 2004 | L | N | H | L | L | L | L |
| Zhang Q 2022 | L | L | H | L | L | L | L |
| Yan L 2022 | L | N | H | N | L | L | N |
| Dai G 2016 | L | H | H | N | L | L | N |

**Additional file 4.** Pairwise Meta-Analysis.

FMA(1 vs 16)

| Study or Subgroup | Experimental Mean | Experimental SD | Experimental Total | Control Mean | Control SD | Control Total | Weight | Std. Mean Difference IV, Fixed, 95% CI |
|---|---|---|---|---|---|---|---|---|
| Wang Y 2022 | 6.97 | 2.37 | 60 | 5.12 | 2.08 | 60 | 100.0% | 0.82 [0.45, 1.20] |
| **Total (95% CI)** | | | **60** | | | **60** | **100.0%** | **0.82 [0.45, 1.20]** |

Heterogeneity: Not applicable
Test for overall effect: Z = 4.33 (P < 0.0001)

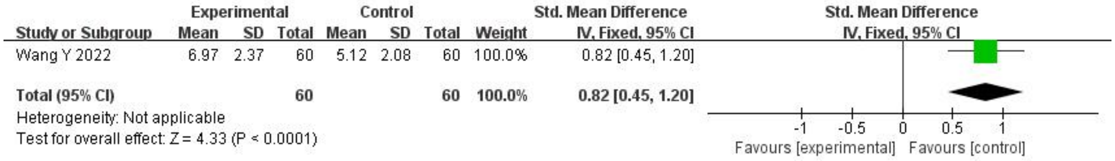


FMA(2 vs 3)

| Study or Subgroup | Experimental Mean | Experimental SD | Experimental Total | Control Mean | Control SD | Control Total | Weight | Std. Mean Difference IV, Fixed, 95% CI |
|---|---|---|---|---|---|---|---|---|
| Zeng Y 2021 | 4.17 | 6.59 | 56 | 5.82 | 6.16 | 56 | 100.0% | -0.26 [-0.63, 0.12] |
| **Total (95% CI)** | | | **56** | | | **56** | **100.0%** | **-0.26 [-0.63, 0.12]** |

Heterogeneity: Not applicable
Test for overall effect: Z = 1.35 (P = 0.18)

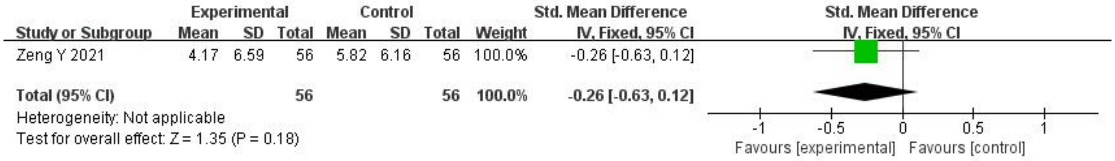


FMA(2 vs 7)

| Study or Subgroup | Experimental Mean | Experimental SD | Experimental Total | Control Mean | Control SD | Control Total | Weight | Std. Mean Difference IV, Fixed, 95% CI |
|---|---|---|---|---|---|---|---|---|
| Wu L 2021 | 9.63 | 4.96 | 67 | 5.56 | 4.53 | 67 | 100.0% | 0.85 [0.50, 1.21] |
| **Total (95% CI)** | | | **67** | | | **67** | **100.0%** | **0.85 [0.50, 1.21]** |

Heterogeneity: Not applicable
Test for overall effect: Z = 4.72 (P < 0.00001)

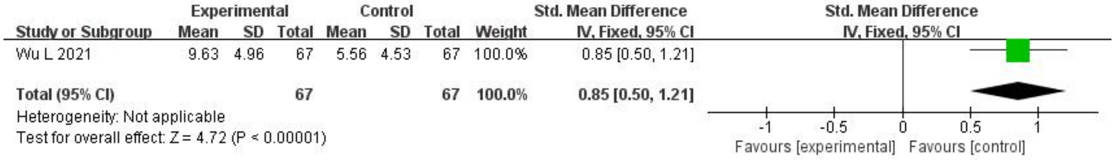


FMA(4 vs 16)

| Study or Subgroup | Experimental Mean | Experimental SD | Experimental Total | Control Mean | Control SD | Control Total | Weight | Std. Mean Difference IV, Random, 95% CI |
|---|---|---|---|---|---|---|---|---|
| Cui J 2020 | 15.5 | 7.19 | 35 | 6.71 | 5.37 | 35 | 48.3% | 1.37 [0.85, 1.89] |
| Yin G 2021 | 2.53 | 1.51 | 36 | 1.29 | 1.52 | 36 | 51.7% | 0.81 [0.33, 1.29] |
| **Total (95% CI)** | | | **71** | | | **71** | **100.0%** | **1.08 [0.53, 1.63]** |

Heterogeneity: $Tau^2 = 0.09$; $Chi^2 = 2.38$, df = 1 (P = 0.12); $I^2 = 58\%$
Test for overall effect: Z = 3.86 (P = 0.0001)

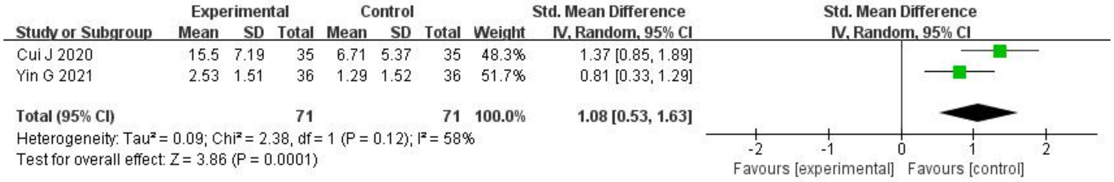

FMA(5 vs 16)

| | Experimental | | | Control | | | | Std. Mean Difference |
|---|---|---|---|---|---|---|---|---|
| Study or Subgroup | Mean | SD | Total | Mean | SD | Total | Weight | IV, Fixed, 95% CI |
| Huang Z 2018 | 2.58 | 1.63 | 40 | 1.08 | 2.1 | 40 | 100.0% | 0.79 [0.33, 1.25] |
| **Total (95% CI)** | | | **40** | | | **40** | **100.0%** | **0.79 [0.33, 1.25]** |

Heterogeneity: Not applicable
Test for overall effect: Z = 3.40 (P = 0.0007)

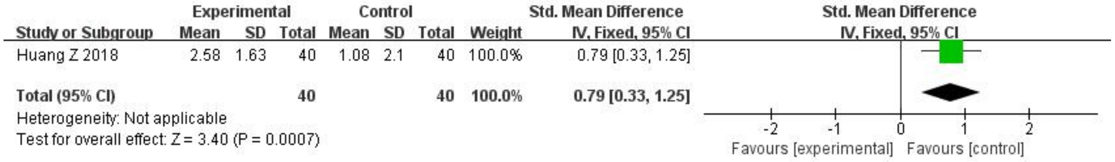


FMA(6 vs 16)

| | Experimental | | | Control | | | | Std. Mean Difference |
|---|---|---|---|---|---|---|---|---|
| Study or Subgroup | Mean | SD | Total | Mean | SD | Total | Weight | IV, Fixed, 95% CI |
| Zhang L 2021 | 8.74 | 4.47 | 30 | 6.46 | 4.55 | 30 | 100.0% | 0.50 [-0.02, 1.01] |
| **Total (95% CI)** | | | **30** | | | **30** | **100.0%** | **0.50 [-0.02, 1.01]** |

Heterogeneity: Not applicable
Test for overall effect: Z = 1.90 (P = 0.06)

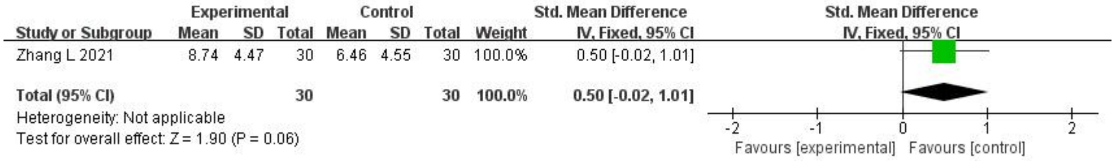


FMA(7 vs 9)

| | Experimental | | | Control | | | | Std. Mean Difference |
|---|---|---|---|---|---|---|---|---|
| Study or Subgroup | Mean | SD | Total | Mean | SD | Total | Weight | IV, Fixed, 95% CI |
| Zhao M 2018 | 15.23 | 1.44 | 30 | 17.6 | 1.57 | 30 | 100.0% | -1.55 [-2.13, -0.97] |
| **Total (95% CI)** | | | **30** | | | **30** | **100.0%** | **-1.55 [-2.13, -0.97]** |

Heterogeneity: Not applicable
Test for overall effect: Z = 5.23 (P < 0.00001)

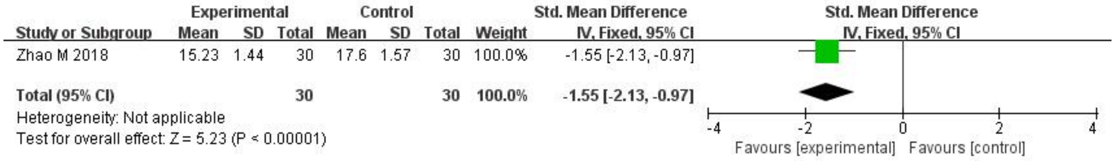


FMA(7 vs 14)

| | Experimental | | | Control | | | | Std. Mean Difference |
|---|---|---|---|---|---|---|---|---|
| Study or Subgroup | Mean | SD | Total | Mean | SD | Total | Weight | IV, Fixed, 95% CI |
| Wang Y 2015 | 6.54 | 2.59 | 60 | 7.76 | 2.56 | 58 | 100.0% | -0.47 [-0.84, -0.10] |
| **Total (95% CI)** | | | **60** | | | **58** | **100.0%** | **-0.47 [-0.84, -0.10]** |

Heterogeneity: Not applicable
Test for overall effect: Z = 2.52 (P = 0.01)

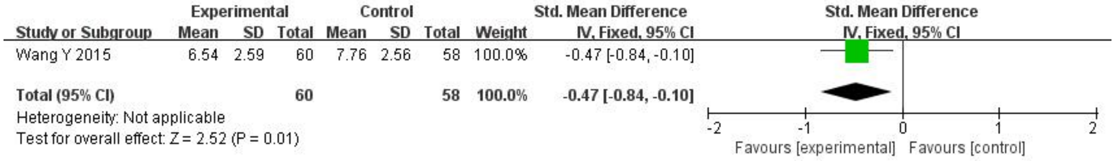


FMA(7 vs 15)

| | Experimental | | | Control | | | | Std. Mean Difference |
|---|---|---|---|---|---|---|---|---|
| Study or Subgroup | Mean | SD | Total | Mean | SD | Total | Weight | IV, Fixed, 95% CI |
| Wang Y 2019 | 14.2 | 7.57 | 56 | 5.53 | 6.76 | 56 | 100.0% | 1.20 [0.80, 1.60] |
| **Total (95% CI)** | | | **56** | | | **56** | **100.0%** | **1.20 [0.80, 1.60]** |

Heterogeneity: Not applicable
Test for overall effect: Z = 5.83 (P < 0.00001)

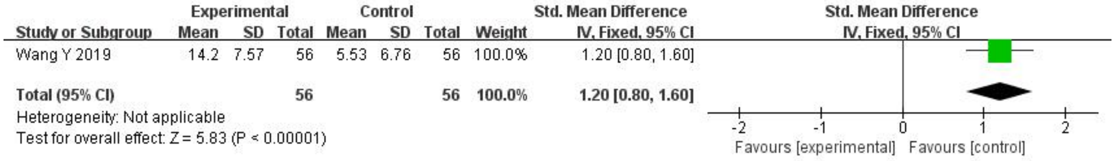

FMA(7 vs 16)

| Study or Subgroup | Experimental Mean | Experimental SD | Experimental Total | Control Mean | Control SD | Control Total | Weight | Std. Mean Difference IV, Random, 95% CI |
|---|---|---|---|---|---|---|---|---|
| Chen J 2018 | 14.93 | 3.18 | 52 | 9.96 | 2.83 | 52 | 10.4% | 1.64 [1.19, 2.09] |
| Dang H 2019 | 9.92 | 2.78 | 50 | 3.34 | 2.83 | 50 | 9.8% | 2.33 [1.82, 2.84] |
| David N. Alexander 2004 | 4.6 | 7.86 | 16 | 0.9 | 3.9 | 15 | 7.9% | 0.57 [-0.15, 1.30] |
| He X 2008 | 9.32 | 3.98 | 30 | 7.33 | 4.32 | 30 | 9.8% | 0.47 [-0.04, 0.99] |
| Qi Y 2016 | 13.12 | 3.22 | 49 | 10.99 | 2.71 | 49 | 10.7% | 0.71 [0.30, 1.12] |
| Sun S 2020 | 2.63 | 1.87 | 54 | 1.16 | 1.72 | 54 | 10.9% | 0.81 [0.42, 1.21] |
| Tan S 2018 | 15.2 | 3.48 | 44 | 10.71 | 2.88 | 44 | 10.2% | 1.39 [0.93, 1.86] |
| Wang Y 2019 | 14.2 | 7.57 | 56 | 6.52 | 7.06 | 56 | 10.9% | 1.04 [0.65, 1.44] |
| Yin Z 2013 | 15.09 | 3.13 | 31 | 11.39 | 2.95 | 30 | 9.5% | 1.20 [0.65, 1.75] |
| Zhu G 2020 | 13.21 | 6.61 | 35 | 5.92 | 6.25 | 35 | 9.9% | 1.12 [0.61, 1.63] |
| **Total (95% CI)** | | | **417** | | | **415** | **100.0%** | **1.14 [0.81, 1.46]** |

Heterogeneity: $Tau^2 = 0.22$; $Chi^2 = 42.62$, df = 9 ($P < 0.00001$); $I^2 = 79\%$
Test for overall effect: Z = 6.78 ($P < 0.00001$)

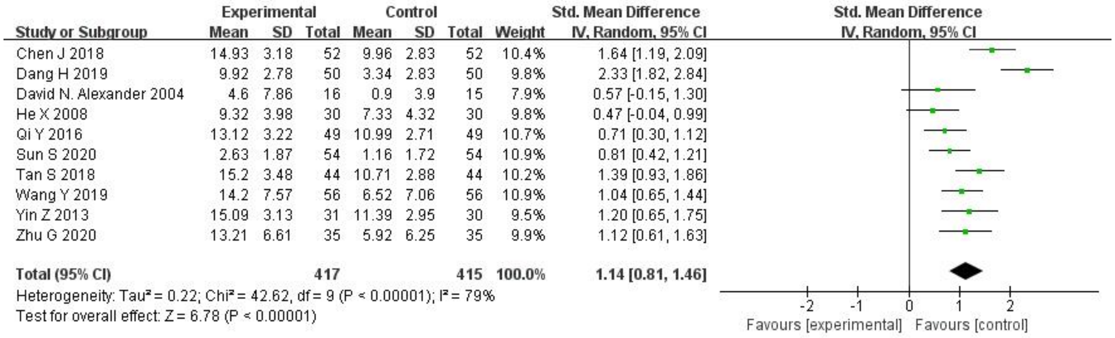


FMA(8 vs 9)

| Study or Subgroup | Experimental Mean | Experimental SD | Experimental Total | Control Mean | Control SD | Control Total | Weight | Std. Mean Difference IV, Random, 95% CI |
|---|---|---|---|---|---|---|---|---|
| Li D 2020 | 11.21 | 3.94 | 41 | 14.92 | 4.07 | 41 | 52.6% | -0.92 [-1.37, -0.46] |
| Wang X 2017 | 2.35 | 4.69 | 39 | 8.14 | 3.84 | 39 | 47.4% | -1.34 [-1.83, -0.84] |
| **Total (95% CI)** | | | **80** | | | **80** | **100.0%** | **-1.12 [-1.53, -0.71]** |

Heterogeneity: $Tau^2 = 0.03$; $Chi^2 = 1.50$, df = 1 ($P = 0.22$); $I^2 = 33\%$
Test for overall effect: Z = 5.32 ($P < 0.00001$)

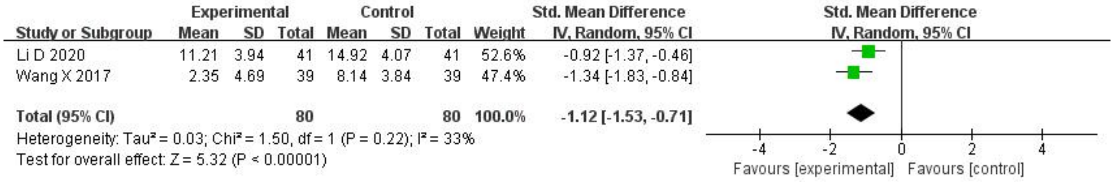


FMA(8 vs 16)

| Study or Subgroup | Experimental Mean | Experimental SD | Experimental Total | Control Mean | Control SD | Control Total | Weight | Std. Mean Difference IV, Random, 95% CI |
|---|---|---|---|---|---|---|---|---|
| Dai G 2016 | 10 | 4.35 | 57 | 5.2 | 4.86 | 57 | 20.3% | 1.03 [0.64, 1.43] |
| Gong W 2015 | 9.51 | 2.79 | 40 | 4 | 2.7 | 40 | 19.0% | 1.99 [1.45, 2.53] |
| Hao Q 2020 | 6.82 | 2.54 | 36 | 4.32 | 2.84 | 36 | 19.5% | 0.92 [0.43, 1.40] |
| Xu Y 2018 | 9.17 | 2.03 | 41 | 6.78 | 1.69 | 43 | 19.6% | 1.27 [0.80, 1.74] |
| Yan L 2022 | 4.8 | 2.91 | 184 | 4.2 | 3.1 | 183 | 21.5% | 0.20 [-0.01, 0.40] |
| **Total (95% CI)** | | | **358** | | | **359** | **100.0%** | **1.06 [0.42, 1.70]** |

Heterogeneity: $Tau^2 = 0.48$; $Chi^2 = 53.62$, df = 4 ($P < 0.00001$); $I^2 = 93\%$
Test for overall effect: Z = 3.26 ($P = 0.001$)

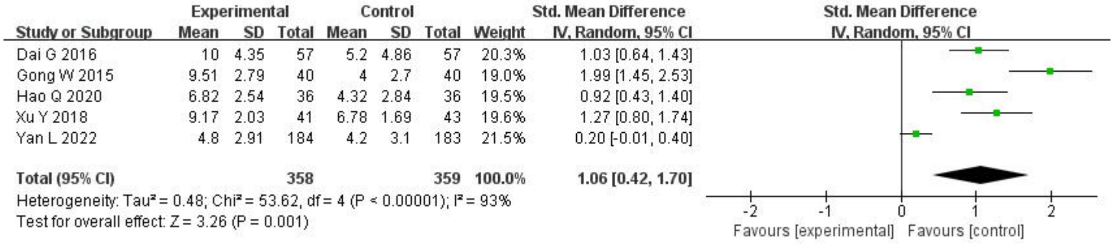


FMA(9 vs 16)

| Study or Subgroup | Experimental Mean | Experimental SD | Experimental Total | Control Mean | Control SD | Control Total | Weight | Std. Mean Difference IV, Fixed, 95% CI |
|---|---|---|---|---|---|---|---|---|
| Hou X 2018 | 14.69 | 4.22 | 48 | 9.41 | 3.81 | 48 | 58.3% | 1.30 [0.86, 1.74] |
| Wang N 2015 | 7.06 | 4.9 | 30 | 3 | 6.37 | 30 | 41.7% | 0.71 [0.18, 1.23] |
| **Total (95% CI)** | | | **78** | | | **78** | **100.0%** | **1.05 [0.72, 1.39]** |

Heterogeneity: $Chi^2 = 2.93$, df = 1 ($P = 0.09$); $I^2 = 66\%$
Test for overall effect: Z = 6.12 ($P < 0.00001$)

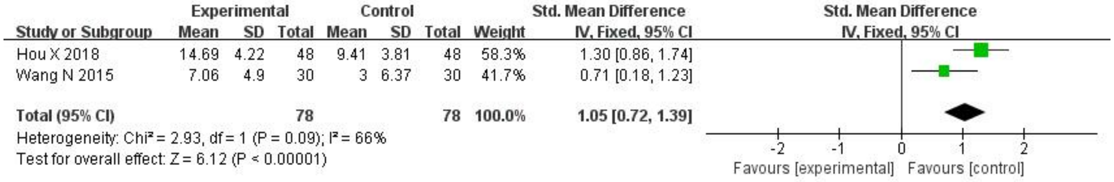

FMA(10 vs 16)

| Study or Subgroup | Experimental Mean | Experimental SD | Experimental Total | Control Mean | Control SD | Control Total | Weight | Std. Mean Difference IV, Random, 95% CI |
|---|---|---|---|---|---|---|---|---|
| Li Z 2016 | 18.54 | 7.29 | 30 | 7.93 | 6.09 | 30 | 18.5% | 1.56 [0.98, 2.14] |
| Wang H 2019 | 11 | 6.82 | 30 | 3.9 | 1.4 | 29 | 18.6% | 1.41 [0.84, 1.99] |
| Xu S 2016 | 9.22 | 4.91 | 36 | 6.88 | 4.29 | 35 | 19.9% | 0.50 [0.03, 0.97] |
| Zeng Y 2016 | 11.46 | 6.27 | 50 | 10.1 | 6.5 | 50 | 20.9% | 0.21 [-0.18, 0.60] |
| Zhu Y 2013 | 13.86 | 7.74 | 98 | 12.84 | 8.3 | 90 | 22.0% | 0.13 [-0.16, 0.41] |
| **Total (95% CI)** | | | **244** | | | **234** | **100.0%** | **0.72 [0.18, 1.27]** |

Heterogeneity: Tau² = 0.33; Chi² = 30.93, df = 4 (P < 0.00001); I² = 87%
Test for overall effect: Z = 2.60 (P = 0.009)

Std. Mean Difference IV, Random, 95% CI

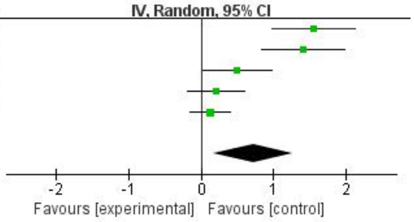


FMA(12 vs 16)

| Study or Subgroup | Experimental Mean | Experimental SD | Experimental Total | Control Mean | Control SD | Control Total | Weight | Std. Mean Difference IV, Fixed, 95% CI |
|---|---|---|---|---|---|---|---|---|
| Yang R 2017 | 9.11 | 4.81 | 30 | 4.88 | 4.81 | 30 | 100.0% | 0.87 [0.34, 1.40] |
| **Total (95% CI)** | | | **30** | | | **30** | **100.0%** | **0.87 [0.34, 1.40]** |

Heterogeneity: Not applicable
Test for overall effect: Z = 3.20 (P = 0.001)

Std. Mean Difference IV, Fixed, 95% CI

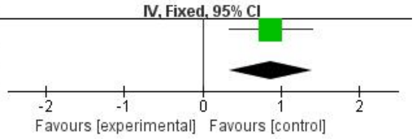


FMA(13 vs 16)

| Study or Subgroup | Experimental Mean | Experimental SD | Experimental Total | Control Mean | Control SD | Control Total | Weight | Std. Mean Difference IV, Fixed, 95% CI |
|---|---|---|---|---|---|---|---|---|
| Chen D 2017 | 7.21 | 6.16 | 48 | 4.46 | 6.03 | 48 | 100.0% | 0.45 [0.04, 0.85] |
| **Total (95% CI)** | | | **48** | | | **48** | **100.0%** | **0.45 [0.04, 0.85]** |

Heterogeneity: Not applicable
Test for overall effect: Z = 2.16 (P = 0.03)

Std. Mean Difference IV, Fixed, 95% CI

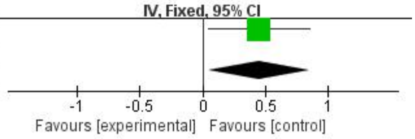


FMA(15 vs 16)

| Study or Subgroup | Experimental Mean | Experimental SD | Experimental Total | Control Mean | Control SD | Control Total | Weight | Std. Mean Difference IV, Fixed, 95% CI |
|---|---|---|---|---|---|---|---|---|
| Wang Y 2019 | 5.53 | 6.76 | 56 | 6.52 | 7.06 | 56 | 100.0% | -0.14 [-0.51, 0.23] |
| **Total (95% CI)** | | | **56** | | | **56** | **100.0%** | **-0.14 [-0.51, 0.23]** |

Heterogeneity: Not applicable
Test for overall effect: Z = 0.75 (P = 0.45)

Std. Mean Difference IV, Fixed, 95% CI

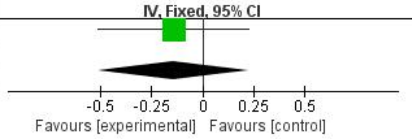


MBI(1 vs 16)

| Study or Subgroup | Experimental Mean | Experimental SD | Experimental Total | Control Mean | Control SD | Control Total | Weight | Std. Mean Difference IV, Fixed, 95% CI |
|---|---|---|---|---|---|---|---|---|
| Wang Y 2022 | 20.25 | 7.83 | 60 | 11 | 7.09 | 60 | 100.0% | 1.23 [0.84, 1.62] |
| **Total (95% CI)** | | | **60** | | | **60** | **100.0%** | **1.23 [0.84, 1.62]** |

Heterogeneity: Not applicable
Test for overall effect: Z = 6.16 (P < 0.00001)

Std. Mean Difference IV, Fixed, 95% CI

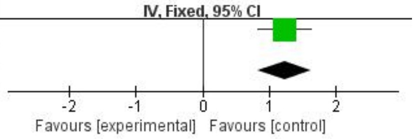


MBI(4 vs 8)

| Study or Subgroup | Experimental Mean | Experimental SD | Experimental Total | Control Mean | Control SD | Control Total | Weight | Std. Mean Difference IV, Fixed, 95% CI |
|---|---|---|---|---|---|---|---|---|
| Tang Z 2012 | 44.9 | 17.11 | 60 | 18.9 | 12.59 | 60 | 100.0% | 1.72 [1.30, 2.14] |
| **Total (95% CI)** | | | **60** | | | **60** | **100.0%** | **1.72 [1.30, 2.14]** |

Heterogeneity: Not applicable
Test for overall effect: Z = 8.01 (P < 0.00001)

Std. Mean Difference IV, Fixed, 95% CI

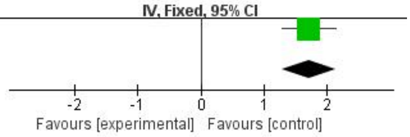

MBI(4 vs 16)

| Study or Subgroup | Experimental Mean | Experimental SD | Experimental Total | Control Mean | Control SD | Control Total | Weight | Std. Mean Difference IV, Fixed, 95% CI |
|---|---|---|---|---|---|---|---|---|
| Yin G 2021 | 29.88 | 7.14 | 36 | 23.44 | 7.91 | 36 | 100.0% | 0.85 [0.36, 1.33] |
| **Total (95% CI)** | | | **36** | | | **36** | **100.0%** | **0.85 [0.36, 1.33]** |

Heterogeneity: Not applicable
Test for overall effect: Z = 3.43 (P = 0.0006)

Std. Mean Difference
IV, Fixed, 95% CI

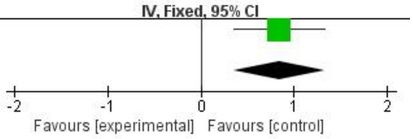


MBI(5 vs 16)

| Study or Subgroup | Experimental Mean | Experimental SD | Experimental Total | Control Mean | Control SD | Control Total | Weight | Std. Mean Difference IV, Fixed, 95% CI |
|---|---|---|---|---|---|---|---|---|
| Huang Z 2018 | 28.24 | 5.69 | 40 | 23.83 | 6.56 | 40 | 100.0% | 0.71 [0.26, 1.16] |
| **Total (95% CI)** | | | **40** | | | **40** | **100.0%** | **0.71 [0.26, 1.16]** |

Heterogeneity: Not applicable
Test for overall effect: Z = 3.08 (P = 0.002)

Std. Mean Difference
IV, Fixed, 95% CI

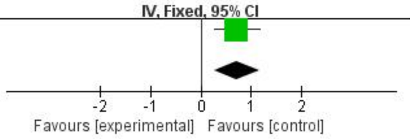


MBI(6 vs 16)

| Study or Subgroup | Experimental Mean | Experimental SD | Experimental Total | Control Mean | Control SD | Control Total | Weight | Std. Mean Difference IV, Fixed, 95% CI |
|---|---|---|---|---|---|---|---|---|
| Zhang L 2021 | 26.17 | 15.21 | 30 | 17.5 | 14.26 | 30 | 100.0% | 0.58 [0.06, 1.10] |
| **Total (95% CI)** | | | **30** | | | **30** | **100.0%** | **0.58 [0.06, 1.10]** |

Heterogeneity: Not applicable
Test for overall effect: Z = 2.20 (P = 0.03)

Std. Mean Difference
IV, Fixed, 95% CI

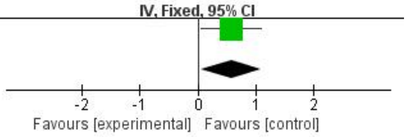


MBI(7 vs 14)

| Study or Subgroup | Experimental Mean | Experimental SD | Experimental Total | Control Mean | Control SD | Control Total | Weight | Std. Mean Difference IV, Fixed, 95% CI |
|---|---|---|---|---|---|---|---|---|
| Wang Y 2015 | 2.28 | 7.04 | 58 | 8.62 | 2.73 | 60 | 100.0% | -1.19 [-1.58, -0.79] |
| **Total (95% CI)** | | | **58** | | | **60** | **100.0%** | **-1.19 [-1.58, -0.79]** |

Heterogeneity: Not applicable
Test for overall effect: Z = 5.93 (P < 0.00001)

Std. Mean Difference
IV, Fixed, 95% CI

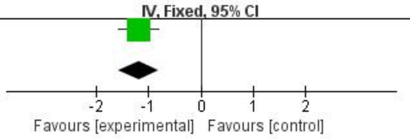


MBI(7 vs 15)

| Study or Subgroup | Experimental Mean | Experimental SD | Experimental Total | Control Mean | Control SD | Control Total | Weight | Std. Mean Difference IV, Fixed, 95% CI |
|---|---|---|---|---|---|---|---|---|
| Xu L 2017 | 23.27 | 8.33 | 30 | 9.4 | 10.33 | 30 | 100.0% | 1.46 [0.89, 2.03] |
| **Total (95% CI)** | | | **30** | | | **30** | **100.0%** | **1.46 [0.89, 2.03]** |

Heterogeneity: Not applicable
Test for overall effect: Z = 4.99 (P < 0.00001)

Std. Mean Difference
IV, Fixed, 95% CI

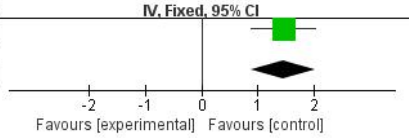

MBI(7 vs 16)

| Study or Subgroup | Experimental Mean | Experimental SD | Experimental Total | Control Mean | Control SD | Control Total | Weight | Std. Mean Difference IV, Random, 95% CI |
|---|---|---|---|---|---|---|---|---|
| Chen J 2018 | 59.79 | 8.38 | 52 | 46 | 9.77 | 52 | 17.5% | 1.50 [1.07, 1.94] |
| Dang H 2019 | 25.86 | 7.2 | 50 | 11.02 | 7.08 | 50 | 16.5% | 2.06 [1.57, 2.55] |
| Sun S 2020 | 34.77 | 20.17 | 54 | 20.5 | 19.03 | 54 | 18.4% | 0.72 [0.33, 1.11] |
| Tan S 2018 | 34.35 | 10.18 | 44 | 21.59 | 9.12 | 44 | 17.0% | 1.31 [0.85, 1.77] |
| Xu L 2017 | 23.27 | 8.33 | 30 | 12.53 | 7.21 | 30 | 15.1% | 1.36 [0.80, 1.93] |
| Yin Z 2013 | 32.87 | 5.6 | 31 | 24.9 | 7.76 | 30 | 15.5% | 1.17 [0.62, 1.71] |
| **Total (95% CI)** | | | **261** | | | **260** | **100.0%** | **1.35 [0.97, 1.72]** |

Heterogeneity: $Tau^2 = 0.16$; $Chi^2 = 18.89$, $df = 5$ ($P = 0.002$); $I^2 = 74\%$
Test for overall effect: $Z = 6.99$ ($P < 0.00001$)

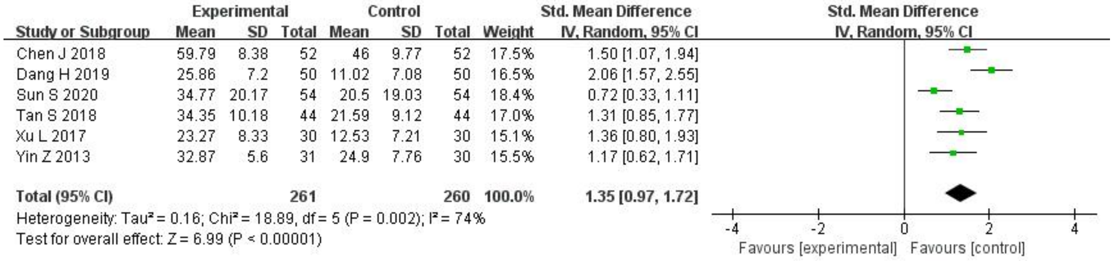


MBI(8 vs 12)

| Study or Subgroup | Experimental Mean | Experimental SD | Experimental Total | Control Mean | Control SD | Control Total | Weight | Std. Mean Difference IV, Fixed, 95% CI |
|---|---|---|---|---|---|---|---|---|
| Duan Y 2021 | 11.5 | 16.43 | 57 | 12 | 15.16 | 46 | 100.0% | -0.03 [-0.42, 0.36] |
| **Total (95% CI)** | | | **57** | | | **46** | **100.0%** | **-0.03 [-0.42, 0.36]** |

Heterogeneity: Not applicable
Test for overall effect: $Z = 0.16$ ($P = 0.87$)

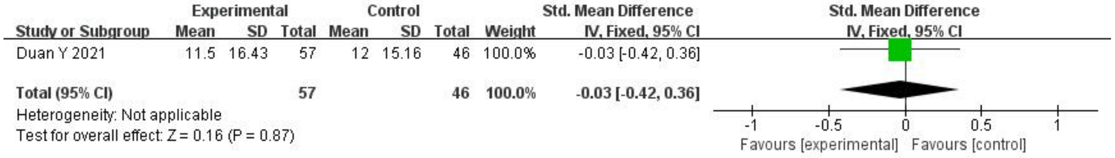


MBI(8 vs 16)

| Study or Subgroup | Experimental Mean | Experimental SD | Experimental Total | Control Mean | Control SD | Control Total | Weight | Std. Mean Difference IV, Fixed, 95% CI |
|---|---|---|---|---|---|---|---|---|
| Hao Q 2020 | 20.03 | 7.88 | 36 | 10.91 | 9.57 | 36 | 100.0% | 1.03 [0.54, 1.52] |
| **Total (95% CI)** | | | **36** | | | **36** | **100.0%** | **1.03 [0.54, 1.52]** |

Heterogeneity: Not applicable
Test for overall effect: $Z = 4.09$ ($P < 0.0001$)

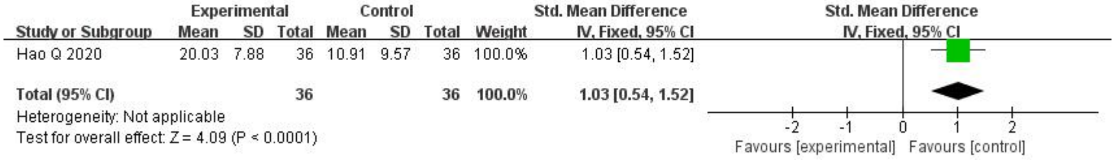


MBI(10 vs 16)

| Study or Subgroup | Experimental Mean | Experimental SD | Experimental Total | Control Mean | Control SD | Control Total | Weight | Std. Mean Difference IV, Fixed, 95% CI |
|---|---|---|---|---|---|---|---|---|
| Wang H 2019 | 23.83 | 5.2 | 30 | 21.89 | 6.47 | 29 | 16.9% | 0.33 [-0.19, 0.84] |
| Zeng Y 2016 | 31.78 | 20.97 | 50 | 23.2 | 25.35 | 50 | 28.6% | 0.37 [-0.03, 0.76] |
| Zhu Y 2013 | 41.58 | 19.25 | 98 | 40.21 | 19.95 | 90 | 54.5% | 0.07 [-0.22, 0.36] |
| **Total (95% CI)** | | | **178** | | | **169** | **100.0%** | **0.20 [-0.01, 0.41]** |

Heterogeneity: $Chi^2 = 1.71$, $df = 2$ ($P = 0.43$); $I^2 = 0\%$
Test for overall effect: $Z = 1.83$ ($P = 0.07$)

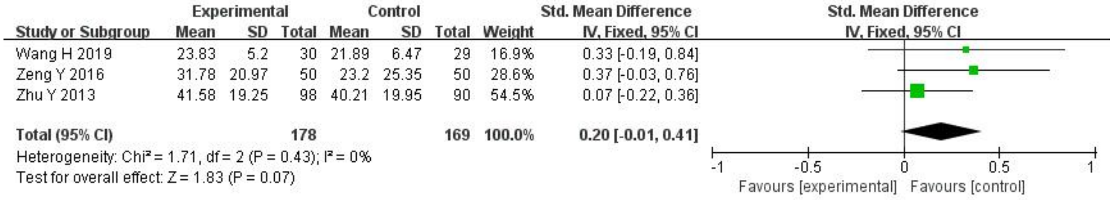


MBI(15 vs 16)

| Study or Subgroup | Experimental Mean | Experimental SD | Experimental Total | Control Mean | Control SD | Control Total | Weight | Std. Mean Difference IV, Fixed, 95% CI |
|---|---|---|---|---|---|---|---|---|
| Xu L 2017 | 9.4 | 10.33 | 30 | 12.53 | 7.21 | 30 | 100.0% | -0.35 [-0.86, 0.16] |
| **Total (95% CI)** | | | **30** | | | **30** | **100.0%** | **-0.35 [-0.86, 0.16]** |

Heterogeneity: Not applicable
Test for overall effect: $Z = 1.33$ ($P = 0.18$)

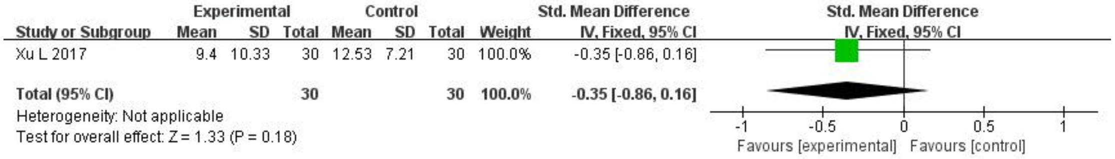

# MBI(16 vs 17)

| Study or Subgroup | Experimental Mean | Experimental SD | Experimental Total | Control Mean | Control SD | Control Total | Weight | Std. Mean Difference IV, Fixed, 95% CI |
|---|---|---|---|---|---|---|---|---|
| You M 2021 | 16 | 8.55 | 26 | 20.82 | 9.47 | 27 | 100.0% | -0.53 [-1.07, 0.02] |
| **Total (95% CI)** | | | **26** | | | **27** | **100.0%** | **-0.53 [-1.07, 0.02]** |
| Heterogeneity: Not applicable | | | | | | | | |
| Test for overall effect: Z = 1.88 (P = 0.06) | | | | | | | | |

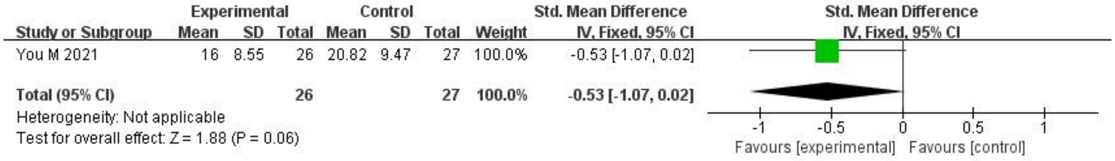


# MAS(4 vs 8)

| Study or Subgroup | Experimental Mean | Experimental SD | Experimental Total | Control Mean | Control SD | Control Total | Weight | Std. Mean Difference IV, Fixed, 95% CI |
|---|---|---|---|---|---|---|---|---|
| Tang Z 2012 | 1.72 | 0.6 | 60 | 1.22 | 0.61 | 60 | 100.0% | 0.82 [0.45, 1.19] |
| **Total (95% CI)** | | | **60** | | | **60** | **100.0%** | **0.82 [0.45, 1.19]** |
| Heterogeneity: Not applicable | | | | | | | | |
| Test for overall effect: Z = 4.31 (P < 0.0001) | | | | | | | | |

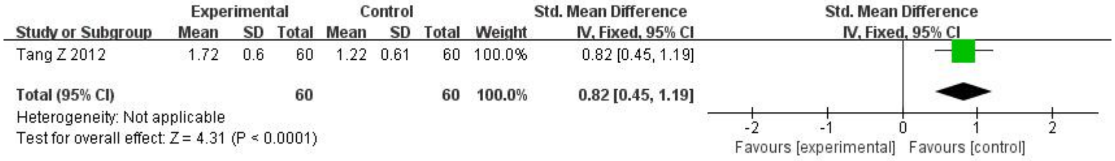


# MAS(6 vs 16)

| Study or Subgroup | Experimental Mean | Experimental SD | Experimental Total | Control Mean | Control SD | Control Total | Weight | Std. Mean Difference IV, Fixed, 95% CI |
|---|---|---|---|---|---|---|---|---|
| Zhang Q 2022 | 1.03 | 0.7 | 31 | 0.57 | 0.93 | 30 | 100.0% | 0.55 [0.04, 1.07] |
| **Total (95% CI)** | | | **31** | | | **30** | **100.0%** | **0.55 [0.04, 1.07]** |
| Heterogeneity: Not applicable | | | | | | | | |
| Test for overall effect: Z = 2.12 (P = 0.03) | | | | | | | | |

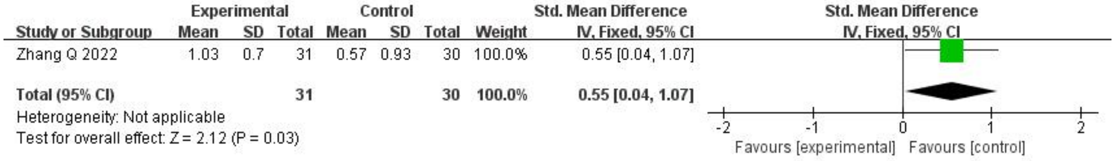


# MAS(7 vs 8)

| Study or Subgroup | Experimental Mean | Experimental SD | Experimental Total | Control Mean | Control SD | Control Total | Weight | Std. Mean Difference IV, Fixed, 95% CI |
|---|---|---|---|---|---|---|---|---|
| Wang X 2017 | 1.07 | 0.3 | 39 | 1.11 | 0.65 | 39 | 100.0% | -0.08 [-0.52, 0.37] |
| **Total (95% CI)** | | | **39** | | | **39** | **100.0%** | **-0.08 [-0.52, 0.37]** |
| Heterogeneity: Not applicable | | | | | | | | |
| Test for overall effect: Z = 0.35 (P = 0.73) | | | | | | | | |

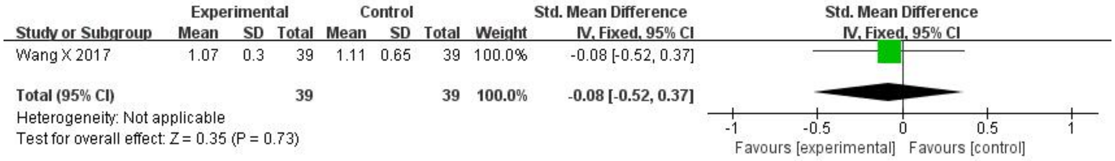


# MAS(7 vs 11)

| Study or Subgroup | Experimental Mean | Experimental SD | Experimental Total | Control Mean | Control SD | Control Total | Weight | Std. Mean Difference IV, Fixed, 95% CI |
|---|---|---|---|---|---|---|---|---|
| Liu C 2020 | 0.22 | 0.58 | 30 | 0.75 | 0.63 | 30 | 100.0% | -0.86 [-1.39, -0.33] |
| **Total (95% CI)** | | | **30** | | | **30** | **100.0%** | **-0.86 [-1.39, -0.33]** |
| Heterogeneity: Not applicable | | | | | | | | |
| Test for overall effect: Z = 3.19 (P = 0.001) | | | | | | | | |

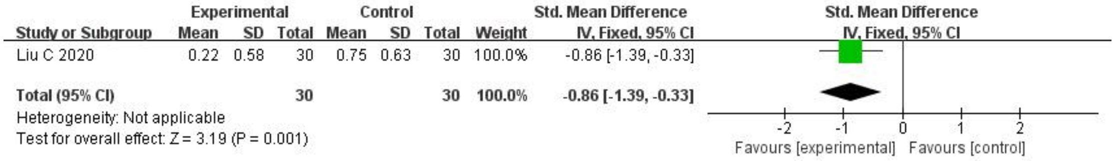

## MAS(7 vs 15)

| Study or Subgroup | Experimental Mean | Experimental SD | Experimental Total | Control Mean | Control SD | Control Total | Weight | Std. Mean Difference IV, Fixed, 95% CI |
|---|---|---|---|---|---|---|---|---|
| Xu L 2017 | 1.97 | 0.82 | 30 | 0.93 | 0.81 | 30 | 100.0% | 1.26 [0.70, 1.82] |
| **Total (95% CI)** | | | **30** | | | **30** | **100.0%** | **1.26 [0.70, 1.82]** |

Heterogeneity: Not applicable
Test for overall effect: Z = 4.43 (P < 0.00001)

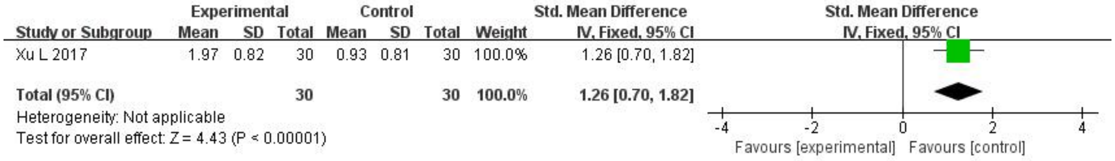


## MAS(7 vs 16)

| Study or Subgroup | Experimental Mean | Experimental SD | Experimental Total | Control Mean | Control SD | Control Total | Weight | Std. Mean Difference IV, Random, 95% CI |
|---|---|---|---|---|---|---|---|---|
| Qi Y 2016 | 1.6 | 0.72 | 49 | 1.24 | 0.69 | 49 | 34.7% | 0.51 [0.10, 0.91] |
| Tan S 2018 | 0.71 | 0.31 | 44 | 0.21 | 0.22 | 44 | 33.2% | 1.84 [1.34, 2.35] |
| Xu L 2017 | 1.97 | 0.82 | 30 | 0.84 | 0.85 | 30 | 32.2% | 1.34 [0.77, 1.90] |
| **Total (95% CI)** | | | **123** | | | **123** | **100.0%** | **1.22 [0.39, 2.05]** |

Heterogeneity: Tau² = 0.47; Chi² = 17.45, df = 2 (P = 0.0002); I² = 89%
Test for overall effect: Z = 2.88 (P = 0.004)

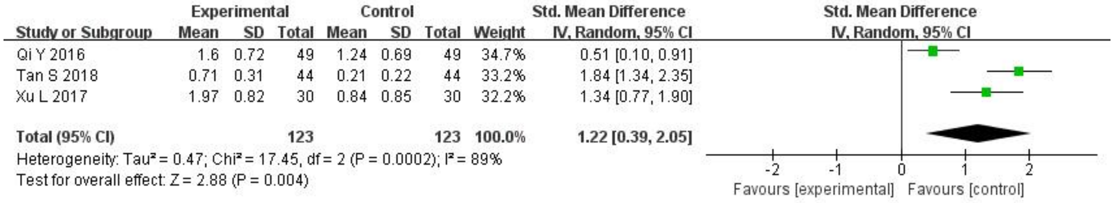


## MAS(8 vs 16)

| Study or Subgroup | Experimental Mean | Experimental SD | Experimental Total | Control Mean | Control SD | Control Total | Weight | Std. Mean Difference IV, Fixed, 95% CI |
|---|---|---|---|---|---|---|---|---|
| Hao Q 2020 | 0.56 | 0.42 | 36 | 0.33 | 0.58 | 36 | 100.0% | 0.45 [-0.02, 0.92] |
| **Total (95% CI)** | | | **36** | | | **36** | **100.0%** | **0.45 [-0.02, 0.92]** |

Heterogeneity: Not applicable
Test for overall effect: Z = 1.88 (P = 0.06)

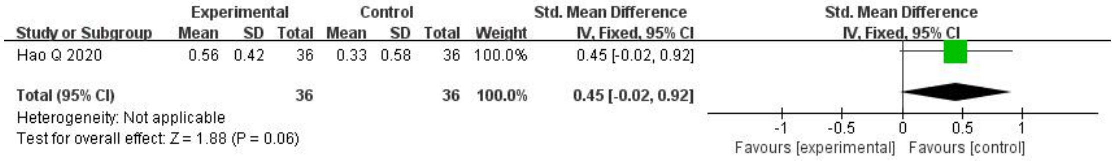


## MAS(10 vs 16)

| Study or Subgroup | Experimental Mean | Experimental SD | Experimental Total | Control Mean | Control SD | Control Total | Weight | Std. Mean Difference IV, Fixed, 95% CI |
|---|---|---|---|---|---|---|---|---|
| Wang H 2019 | 0.7 | 0.49 | 30 | 0.36 | 0.49 | 29 | 100.0% | 0.68 [0.16, 1.21] |
| **Total (95% CI)** | | | **30** | | | **29** | **100.0%** | **0.68 [0.16, 1.21]** |

Heterogeneity: Not applicable
Test for overall effect: Z = 2.55 (P = 0.01)

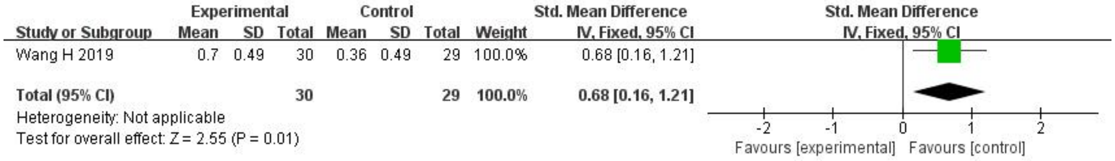


## MAS(15 vs 16)

| Study or Subgroup | Experimental Mean | Experimental SD | Experimental Total | Control Mean | Control SD | Control Total | Weight | Std. Mean Difference IV, Fixed, 95% CI |
|---|---|---|---|---|---|---|---|---|
| Xu L 2017 | 0.93 | 0.81 | 30 | 0.84 | 0.85 | 30 | 100.0% | 0.11 [-0.40, 0.61] |
| **Total (95% CI)** | | | **30** | | | **30** | **100.0%** | **0.11 [-0.40, 0.61]** |

Heterogeneity: Not applicable
Test for overall effect: Z = 0.41 (P = 0.68)

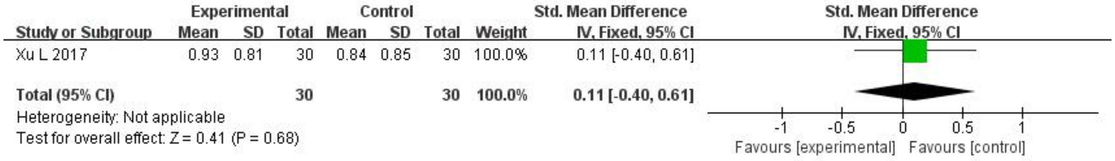

BBS(2 vs 7)

| Study or Subgroup | Experimental Mean | Experimental SD | Experimental Total | Control Mean | Control SD | Control Total | Weight | Std. Mean Difference IV, Fixed, 95% CI |
|---|---|---|---|---|---|---|---|---|
| Wu L 2021 | 13.03 | 6.26 | 67 | 7.13 | 6.31 | 67 | 100.0% | 0.93 [0.58, 1.29] |
| **Total (95% CI)** | | | **67** | | | **67** | **100.0%** | **0.93 [0.58, 1.29]** |

Heterogeneity: Not applicable
Test for overall effect: Z = 5.12 (P < 0.00001)

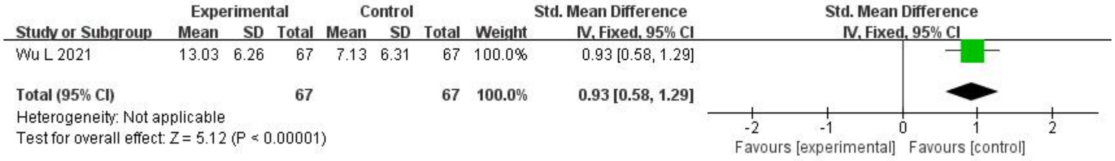


BBS(7 vs 16)

| Study or Subgroup | Experimental Mean | Experimental SD | Experimental Total | Control Mean | Control SD | Control Total | Weight | Std. Mean Difference IV, Random, 95% CI |
|---|---|---|---|---|---|---|---|---|
| Bo F 2021 | 9.92 | 5.57 | 31 | 5.88 | 6.42 | 30 | 16.4% | 0.66 [0.15, 1.18] |
| Dang H 2019 | 19.7 | 3.77 | 50 | 10.9 | 3.26 | 50 | 16.3% | 2.48 [1.95, 3.00] |
| He X 2008 | 17.49 | 7.66 | 30 | 13.88 | 8.13 | 30 | 16.4% | 0.45 [-0.06, 0.96] |
| Song Y 2017 | 16.29 | 6.67 | 80 | 6.17 | 5.02 | 80 | 17.5% | 1.71 [1.34, 2.07] |
| Sun S 2020 | 9.11 | 8.57 | 54 | 2.93 | 7.28 | 54 | 17.3% | 0.77 [0.38, 1.16] |
| Yin Z 2013 | 21.69 | 5.43 | 31 | 14.48 | 6.31 | 30 | 16.1% | 1.21 [0.66, 1.76] |
| **Total (95% CI)** | | | **276** | | | **274** | **100.0%** | **1.21 [0.63, 1.80]** |

Heterogeneity: $Tau^2 = 0.47$; $Chi^2 = 46.98$, df = 5 (P < 0.00001); $I^2 = 89\%$
Test for overall effect: Z = 4.09 (P < 0.0001)

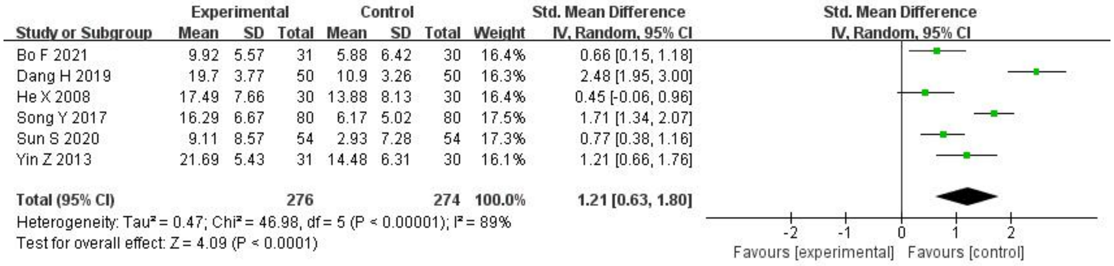


BBS(8 vs 16)

| Study or Subgroup | Experimental Mean | Experimental SD | Experimental Total | Control Mean | Control SD | Control Total | Weight | Std. Mean Difference IV, Fixed, 95% CI |
|---|---|---|---|---|---|---|---|---|
| Hao Q 2020 | 10.28 | 9.26 | 36 | 5.72 | 9.8 | 36 | 54.5% | 0.47 [0.00, 0.94] |
| Xu Y 2018 | 13.55 | 1.9 | 41 | 10.54 | 1.33 | 43 | 45.5% | 1.83 [1.31, 2.34] |
| **Total (95% CI)** | | | **77** | | | **79** | **100.0%** | **1.09 [0.74, 1.44]** |

Heterogeneity: $Chi^2 = 14.57$, df = 1 (P = 0.0001); $I^2 = 93\%$
Test for overall effect: Z = 6.17 (P < 0.00001)

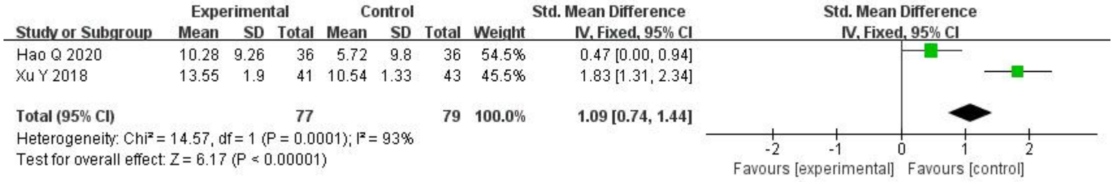

**Additional file 5.** Assessment of inconsistency results.

(1) Node-splitting test result for reduction in FMA-LE score.

| Name | Direct Effect | Indirect Effect | Overall | P-Value |
|---|---|---|---|---|
| 7, 16 | 4.30 (2.43, 6.22) | -0.02 (-4.85, 4.70) | 3.73 (1.92, 5.62) | 0.09 |
| 8, 16 | 3.12 (0.48, 5.83) | 6.23 (1.45, 11.11) | 3.85 (1.52, 6.25) | 0.25 |
| 9, 16 | 4.69 (0.11, 9.42) | 6.95 (2.38, 11.72) | 5.81 (2.56, 9.09) | 0.48 |
| 7, 8 | 5.79 (0.21, 11.41) | -1.26 (-4.08, 1.54) | 0.13 (-2.64, 2.92) | 0.03 |
| 7, 9 | 2.41 (-3.84, 8.71) | 2.00 (-2.37, 6.20) | 2.07 (-1.43, 5.46) | 0.91 |
| 8, 9 | 3.76 (-2.54, 10.06) | 1.12 (-3.30, 5.63) | 1.95 (-1.71, 5.54) | 0.48 |

(2) Node-splitting test result for reduction in MBI score.

| Name | Direct Effect | Indirect Effect | Overall | P-Value |
|---|---|---|---|---|
| 4, 16 | 6.26 (-0.26, 13.08) | 35.15 (24.63, 45.25) | 15.54 (3.19, 28.12) | 0 |
| 8, 16 | 9.09 (2.21, 16.07) | -19.66 (-29.85, -9.43) | -0.30 (-12.58, 12.27) | 0 |
| 4, 8 | -25.95 (-33.78, -18.20) | 2.82 (-7.22, 12.00) | -15.91 (-28.56, -3.40) | 0 |

(3) Node-splitting test result for reduction in MAS score.

| Name | Direct Effect | Indirect Effect | Overall | P-Value |
|---|---|---|---|---|
| 7, 16 | 0.63 (-0.10, 1.43) | 0.18 (-1.68, 2.00) | 0.55 (-0.04, 1.20) | 0.51 |
| 8, 16 | 0.22 (-1.06, 1.52) | 0.66 (-0.80, 2.17) | 0.41 (-0.42, 1.27) | 0.52 |
| 7, 8 | 0.04 (-1.21, 1.27) | -0.36 (-1.89, 1.03) | -0.14 (-0.99, 0.70) | 0.51 |

**Additional file 6.** Assessment of convergence of the model results.

(1)PSRF value in FMA-LE.

| Parameter | PSRF |
|---|---|
| d.16.1 | 1 |
| d.16.10 | 1 |
| d.16.12 | 1 |
| d.16.13 | 1 |
| d.16.4 | 1 |
| d.16.5 | 1 |
| d.16.6 | 1 |
| d.2.3 | 1 |
| d.7.14 | 1 |
| d.7.15 | 1 |
| d.7.16 | 1 |
| d.7.2 | 1 |
| d.7.8 | 1 |
| d.7.9 | 1 |
| sd.d | 1 |

(2) PSRF value in MBI.

| Parameter | PSRF |
|---|---|
| d.16.1 | 1 |
| d.16.10 | 1 |
| d.16.15 | 1 |
| d.16.17 | 1 |
| d.16.4 | 1 |
| d.16.5 | 1 |
| d.16.6 | 1 |
| d.16.7 | 1 |
| d.16.8 | 1 |
| d.7.14 | 1 |
| d.8.12 | 1 |
| sd.d | 1 |

(3) PSRF value in MAS.

| Parameter | PSRF |
|---|---|
| d.16.10 | 1 |
| d.16.15 | 1 |
| d.16.6 | 1 |
| d.16.7 | 1 |
| d.16.8 | 1 |
| d.7.11 | 1 |
| d.8.4 | 1 |
| sd.d | 1 |

(4) PSRF value in BBS.

| Parameter | PSRF |
|---|---|
| d.16.7 | 1 |
| d.16.8 | 1 |
| d.7.2 | 1 |
| sd.d | 1 |
